# Non-rhizobial nodule endophytes improve nodulation, change root exudation pattern and promote the growth of lentil, for prospective application in fallow soil

**DOI:** 10.3389/fpls.2023.1152875

**Published:** 2023-04-11

**Authors:** Sourav Debnath, Subhradeep Chakraborty, Mrinalini Langthasa, Kamlesh Choure, Vivek Agnihotri, Arpit Srivastava, Piyush Kant Rai, Anita Tilwari, D. K. Maheshwari, Piyush Pandey

**Affiliations:** ^1^ Department of Microbiology, Assam University, Silchar, India; ^2^ Department of Biotechnology, AKS University, Satna, India; ^3^ Department of Microbiology, Barkatullah University, Bhopal, India; ^4^ Department of Botany and Microbiology, Gurukula Kangri University, Haridwar, Uttarakhand, India

**Keywords:** lentil (*Lens culinaris*), rhizosphere, NRES, colonization, root metabolites, rhizospheric community structure

## Abstract

Non-rhizobial endophytes (NREs) are active colonizers inhabiting the root nodules. Though their active role in the lentil agroecosystem is not well defined, here we observed that these NREs might promote the growth of lentils, modulate rhizospheric community structure and could be used as promising organisms for optimal use of rice fallow soil. NREs from root nodules of lentils were isolated and examined for plant growth-promoting traits, exopolysaccharide (EPS) and biofilm production, root metabolites, and the presence of nifH and nifK elements. The greenhouse experiment with the chosen NREs, i.e., Serratia plymuthica 33GS and Serratia sp. R6 significantly increased the germination rate, vigour index, development of nodules (in non-sterile soil) and fresh weight of nodules (33GS 94%, R6 61% growth) and length of the shoot (33GS 86%, R6 51.16%) as well as chlorophyll levels when compared to the uninoculated control. Scanning Electron Microscopy (SEM) revealed that both isolates could successfully colonize the roots and elicit root hair growth. The inoculation of the NREs resulted in specific changes in root exudation patterns. The plants with 33GS and R6 treatment significantly stimulated the exudation of triterpenes, fatty acids, and their methyl esters in comparison to the uninoculated plants, altering the rhizospheric microbial community structure. Proteobacteria dominated the rhizospheric microbiota in all the treatments. Treatment with 33GS or R6 also enhanced the relative abundance of other favourable microbes, including Rhizobium, Mesorhizobium, and Bradyrhizobium. The correlation network analysis of relative abundances resulted in numerous bacterial taxa, which were in cooperation with each other, having a possible role in plant growth promotion. The results indicate the significant role of NREs as plant growth promoters, which also includes their role in root exudation patterns, enhancement of soil nutrient status and modulation of rhizospheric microbiota, suggesting their prospects in sustainable, and bio-based agriculture.

## 1 Introduction

Legume-rhizobial symbiosis is known for the fixation of atmospheric nitrogen through a unique structure called root nodules. Root nodules are typical structures harbouring different types of bacteria (Kumar et al., 2017; Dekak et al., 2020), which are predominantly colonized with rhizobial species (Palaniappan et al., 2010), while other bacteria present inside the nodules are collectively known as non-rhizobial endophytes (NREs) (Preyanga et al., 2021). Divergent groups of NREs have been reported to assist the nodulation process and stimulate plant growth (Preyanga et al., 2021). These NREs enter the plant through tissues and maintain themselves in the internal compartment of the plant (Hardoim et al., 2008). Some studies indicate that NREs may enhance plant growth, increase productivity, and protect against pathogens (Vessey, 2003; Saharan and Nehra, 2011; Ge et al., 2023).

It was previously believed that only rhizobial are solely in-charge of symbiotic BNF inside root nodule microenvironments. Recent research suggests that the root nodule contains a greater variety of nodule-associated bacteria (De Meyer et al., 2015). Consequently, different genera of NREs had been reported from various studies, which include*, Stenotrophomonas, Paenibacillus, Arthrobacter, Klebsiella, Pseudomonas, Bacillus, Bosea, Enterobacter, Acinetobacter, Micromonospora, Agrobacterium, Mycobacterium, Phyllobacterium, Pantoea*, and *Serratia* (Vandamme et al., 2002; Valverde et al., 2003; Velázquez et al., 2013; Xu et al., 2014; Dhole et al., 2016; Zaheer et al., 2016; Raja and Uthandi, 2019). The NREs enter the nodule *via* infection threads containing rhizobial isolates and colonize the inner region of root nodules (Pandya et al., 2015; Zgadzaj et al., 2015). Although these bacteria do not induce nodules on their own, they improve nodule formation when co-inoculated with appropriate rhizobia and have many plants growth-promoting properties (Li et al., 2012; Mustaq et al., 2023).

*Lens culinaris* (lentils) are economically significant leguminous crops cultivated worldwide, containing substantial amounts of fibre, protein, amino acids, fatty acids, and trace minerals (Alam et al., 2019). This pulse has been associated with many potential benefits to human health (Danihelová and Šturdík, 2012) and thus has become an essential and inseparable commodity in the human diet. There has been a rapid increase in the consumption of lentils. (Khazaei et al., 2019). Therefore, it is essential to increase lentil production while maximizing the efficient use of land resources, including rice fallow fields. Rice fallow lands are a natural resource that accounts for 162 million ha worldwide, and 140 million ha falls inside Asia (Woodhead and Singh, 2002; Lal et al., 2020; Gautam et al., 2021). Rice monoculture reduces the farmlands’ productivity and constricts the available resource usage. The use of multiple cropping systems, such as rice cultivation in combination with various legume plants, can increase productivity while making efficient use of natural resources and improving soil conditions. (Layek et al., 2018).

Root exudates are chemical signals which hold crucial mechanisms for plant-microbe interactions (Bais et al., 2006). These are a diverse group of compounds that includes 10-50% of fixed plant-carbon, such as fatty acids and their methyl esters, organic acids, amino acids, and flavonoids (Jones et al., 2009; Kuijken et al., 2015; Massalha et al., 2017; Lu et al., 2021). The process of exudation changes with the growth of the root and the process of exudation (Korenblum et al., 2020). Root exudates play a central role in recruiting beneficial microbes for optimum plant growth and help to adapt to various biotic or abiotic factors (Rudrappa et al., 2008; Rajniak et al., 2018; Stringlis et al., 2018). There are few studies on the root exudation pattern of lentils and how these metabolites manipulate the rhizospheric microbiota. These metabolites take part in the below-ground activities in a specific way, but their mechanism and biological role remains uncertain (Dardanelli et al., 2010; Ren et al., 2015; Liu et al., 2017; Ankati et al., 2019; Zhang et al., 2019).

Maintaining the beneficial microbial flora in a typical rice fallow is crucial for the health of the soil and the multipartite interaction between plants and the rhizosphere (Morgan et al., 2005; Das et al., 2022). Therefore, to comprehend the relationship between plant nutrition and crop yields, it is necessary to identify the community structure and its role in plant growth (Verma et al., 2013). Ongoing studies on plant growth-promoting bacteria (PGPB) has revealed successful modulation of the rhizospheric bacterial community (Abbasi et al., 2021; Kaur et al., 2022). It has been reported that utilizing rhizobial isolates can manipulate the dynamics of the microbial community in the rhizosphere, leading to improved growth of lentils and soil quality (Debnath et al., 2023).

**Figure 1:**
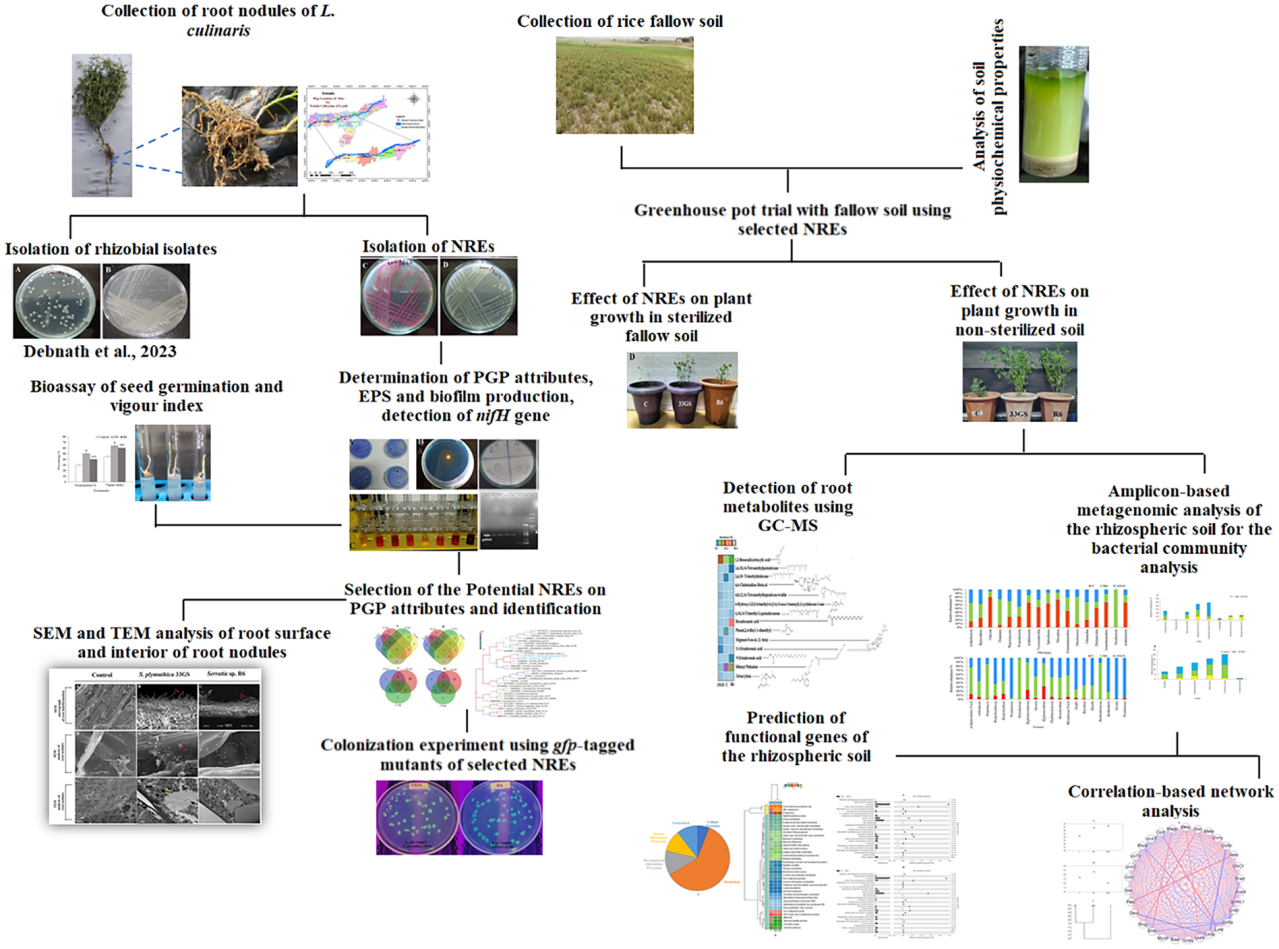
Schematic flow chart of the methodologies used in the study.

The most crucial factor in enhancing *L. culinaris* growth is understanding the microbiome of the lentil and the interactions between microbes and the rhizosphere in the fallow soil of rice, which has not been investigated previously. Furthermore, due to the primary focus on rhizobacteria and rhizobia, the potential contribution of NREs is not yet evident. Although there have been several reports about the NREs’ presence, the possibility of microbiota from lentils is not fully understood (Geetha Thanuja et al., 2020). Therefore, the present research has been undertaken with the objectives - i) to evaluate the potential of NREs to enhance lentil plant growth in fallow soil and investigate their effect on the microbial community in the rhizosphere; ii) to evaluate NREs colonization; iii) and also to determine any variation in root exudation pattern of host plant; with aim to promote the optimal utilization of rice fallow lands for lentil cultivation. **Figure 1** illustrates a flow chart that depicts the methods utilized in this research.

## 2 Material and methods

### 2.1 Collection of lentil root nodules

Nodules from the root of *L. culinaris* were obtained from five different lentil-growing regions of Assam, India, namely Goalpara (26.14722 N, 90.65107 E), Lakhipur (26.03576 N, 90.38364 E), Nagaon (26.3834 N, 92.6469 E), Jakhlabandha (26.59046 N, 92.99376 E), and Goroimari (26.101636 N, 91.313636 E) (**Supplementary Figure 1**). Five plants were randomly picked from each location. The lentil plants were 35-45 days old when the nodules were collected, which occurred after the flowers appeared on the plant. Root nodules from healthy plants were collected and transported to the lab in liquid nitrogen for further studies.

### 2.2 Isolation and molecular characterization of the NREs

The surface of the root nodules was treated with 70% ethanol solution for 30 sec, with subsequent treatment of 0.1% NaOCl for 30 sec_;_ followed by six washes with sterile distilled water. The nodules were crushed with sterile forceps in 1.5 mL microfuge tubes containing 0.5 mL Yeast Extract Mannitol (YEM) broth and poured onto YEM agar plates for 24-72 h at 28 ± 2°C. The isolated colonies were sub-cultured in plates containing YEM agar medium and preserved at 4°C.

Bacterial isolates were grown in conical flasks containing YEM broth. 5 ml of cells were pelleted from each bacterial suspension, and 50 µl of cell pellets were transferred into 1.5 microcentrifuge tubes (Cooper et al., 1998). A Hi-media Bacterial Genomic DNA Extraction kit was used to isolate the total genomic DNA. The partial 16S rRNA gene was amplified using the primers 27F (5´-GAGAGTTTGATCCTGGCTCAG-3´) and 1107R (5´-GCTCGTTGCGGGACTTAACC-3´) in a reaction mixture of 30µl. This mixture contained 1.5 µl of template DNA, 2µl of each forward and reverse primers (27F and 1107R), 15µl of Taq 2X master mix, and 9.5 µl of nuclease-free water. The reaction consisted of an initial denaturation at 94°C for 5min, followed by 30 cycles at 94°C for 1min each, 54°C for 1min, and 72°C for 2min. Finally, there was a final extension stage for 10min at 72°C. Sanger’s method was used to sequence the amplicon (Ayyaz et al., 2016), while the sequences were analyzed with DNA baser (v2.9.54). The phylogenetic tree of the sequences was constructed using the Tamura-Nei framework, through neighbourhood merging in MEGA 7 (Tamura and Nei, 1993).

### 2.3 Determination of the plant growth-promoting attributes of NREs

#### 2.3.1 Indole acetic production production

The NREs isolates were grown in YEM broth amended with 2 mg/ml tryptophan for 5 days at 30°C. The cells were pelleted out through centrifugation (15000xg 10 min) and supernatant was mixed with Salkowski reagent (Gordon and Weber, 1951). The absorbance was measured at 530 nm (Devi et al., 2016).

#### 2.3.2 Phosphate solubilization

NREs were inoculated in NBRIP medium and grown at 30°C (Nautiyal, 1999; Kotoky et al., 2017). The supernatant (15000xg 10 min) from 24-h aliquots of 5 mL was separated for analysis and estimated for phosphate concentration as described (Koenig and Johnson, 1942).

#### 2.3.3 Siderophore production

Siderophore production was detected using the Schwyn and Neilands method (Schwyn and Neilands, 1987). Isolates were cultured in an iron-limiting broth to measure the amount of siderophore produced. The supernatant was collected and quantified by mixing it with CAS reagent and absorbance was measured at 630 nm (Alexander and Zuberer, 1991; Payne, 1994).

#### 2.3.4 Amino-cyclopropane-1- carboxylic acid deaminase activity

The NREs were inoculated onto DF salt plates with 3 mM ACC (Dworkin and Foster, 1958; Penrose and Glick, 2003) and incubated for three days at 28°C to observe the growth. The enzyme was quantified by estimating the α-ketobutyrate production using Honma and Shimomura’s method (1978) at 540 nm. The Bradford method (1976) was used to estimate the total protein and activity was expressed as α-ketobutyrate released per milligram of cellular protein per hour.

#### 2.3.5 Hydrogen cyanide production

NREs were grown on glycine-containing nutrient agar plates to evaluate HCN production. A Whatman filter paper soaked in 2% sodium carbonate and 0.5% picric acid was placed over the agar plate and sealed and observed for HCN (Bakker and Schippers, 1987).

#### 2.3.6 Ammonia production

The isolates were cultured in peptone broth and kept at 28 ± 2°C for 48 to 72 hours. A bacterial suspension was created and then subjected to Nessler’s reagent (0.5 mL). The appearance of a yellow or brown colour was taken as evidence of ammonia production (Cappuccino and Sherman, 1992).

#### 2.3.7 Potassium solubilization

Ability to solubilize potassium by the NREs was checked using Aleksandrov medium. The plates were incubated at 30°C for 7 days after inoculation. Colonies with a solubilization zone on the medium were noted to be potassium solubilizing bacteria (KSB) (Sun et al., 2020).

#### 2.3.8 Protease activity

The NREs were evaluated for protease production using a plate assay on a medium having gelatin (15.0 g/l), agar (15 g/l), K_2_HPO_4_ (2.0 g/l), Peptone (5.0 g/l), and glucose (1.0 g/l). The clear zone was measured by flooding the plates with a mercuric chloride solution after 24 hours of incubation at 28°C (Abdel Galil, 1992; Sony and Pott, 2016).

### 2.4 Exopolysaccharides and biofilm formation

The isolates were cultured for five days at 30°C in YEM broth. EPS production was measured using Dertli’s protocol (Dertli et al., 2013) and evaluated spectrophotometrically. The test tube method was used to assay the biofilm production with a partial alteration and determined by measuring the absorbance at 570 nm (Feoktistova et al., 2016).

### 2.5 Construction of *gfp* tagged mutants

The isolates were transformed with the *gfp* plasmid pPROBE-TT’ harboured in *Escherichia coli* strain DH5α using the heat shock method (Taylor et al., 1993). The plasmid pPROBE-TT contains a promotor less green fluorescent protein (*gfp*) gene. The transformed cells were cultured in tetracycline-containing Luria-Bertani (LB) broth and screened using a UV transilluminator for visualizing the green fluorescent colonies.

### 2.6 SEM and TEM visualization of the root surface region and endophytic nodule colonization

The *gfp*-tagged mutants were inoculated to the plants along with the late log phase culture of rhizobial isolate *Pararhizobium giardinii* P1 (Debnath et al., 2023) *via* seed bacterization, and planted in sterilized soil. The growth of root hairs on surface and endophytic colonization of selected NREs were assessed using scanning electron microscopy (SEM). The root samples were collected from the greenhouse trials (described later) and fixed in 2% glutaraldehyde for a time interval of 24 hours at 4°C and washed 0.1 M sodium cacodylate buffer (pH 7) and post-fixed in 1% osmium tetroxide for 2 h at 4°C with 0.1 M sodium cacodylate buffer (Gontia-Mishra et al., 2016). The samples were then dried overnight at room temperature after being dehydrated in a graduated series of acetone (50–100%). Using an electronic sputter coater, samples were adhered to aluminium stubs in a double conductivity strip and covered with a thin coating of gold. The samples were evaluated at different magnifications with the help of the microscope (JEOL/JSM-6360). Transmission electron microscopy (TEM) was used to detect the nitrogen-fixing zone in nodules (TEM). The root nodules were fixed, dehydrated with ethanol, embedded in Epoxy resin, and sectioned for microscopic analysis, as described by Witoń et al. (2016). The *gfp-*tagged mutants were recovered as isolated colonies from the interiors of surface-sterilized nodules to assure endophytic colonization. A negative control was experimented in parallel where surface-sterilized intact nodules were cultured to receive no growth of *gfp*-tagged mutants.

### 2.7 Nitrogen-fixing gene amplification and ‘Acetylene reductase assay’ (ARA)

Total genomic DNA was extracted from the NRE isolates using an Invitrogen Genomic DNA kit. Amplify of *nifH* gene were achieved using Zehr-nifHf (5′-TGYGAYCCNAARGCNGA-3′), and Zehr-nifHr (5′-ADNGCCATCATCATCATYTCNCC-3′), primers (Rashid et al., 2012). The amplification was carried out in 50 µl reaction mixtures, each comprising 1 µl of forward and reverse primer (10 M concentration), 25 µl of Taq Green Master Mix (Invitrogen, India), and 22 µl of nuclease-free water at 94°C for 4 min, followed by 30 cycles of 94°C for 30 s, 50°C for 30 s, following 72°C for 45 s, and final extension at 72°C for 10 min. The amplicons were detected on 1% agarose gels containing TBE buffer. For further confirmation, acetylene reductase assay was performed to evaluate the ability of the isolates to fix atmospheric nitrogen (Kaushal and Kaushal, 2015).

### 2.8 Bioassay for seed germination and vigour index

*Serratia* sp. R6 and *S. plymuthica* 33GS NRE isolates were evaluated for their effect on lentil seed germination and seedling vigour. NaOCl (3%) was used to surface-sterilize lentil seeds (10) per treatment (Johnston-Monje and Raizada, 2011). Isolated colonies of *Serratia* sp. R6 or *S. plymuthica* 33GS were cultured for 48 hours in YEM broth followed by the collection of cell pellets which were washed in sterile NaCl solution (0.05 M) by centrifuging at 10,000g for 15 minutes at 4°C. The sterilized seeds were immersed in bacterial cultures of *Serratia* sp. R6 and *S. plymuthica* 33GS for 20 minutes, while the control seeds (uninoculated) were immersed in NaCl (0.05M) and allowed to dry at room temperature. The seeds were redistributed to petri dishes (sterilized) and incubated at 28°C for 5 days. The experiment was performed in triplicate in 3 replications, and the rate of germination, hypocotyl, and root lengths was recorded. The rate of germination and vigour index was measured by the formula:

Germination rate (%)=(number of seeds germinated ÷ total number of seeds) × 100


Vigour index=% germination × total plant length

### 2.9 Greenhouse trial of lentils in fallow soil with the selected NREs

A greenhouse trial was conducted under a controlled environment to examine the effect of the selected isolates on plant growth. Soil from fallow lands was collected and transported in sterile conditions to the lab. The fallow soil used in this study was taken from a rice agricultural field in Silchar, India (24.8333° N, 92.7789° E). This agricultural land has been in practice for rice production for a period longer than 10 years with regular use of a recommended dose of chemical fertilizers. Soil parameters such as N, P, K, and organic C were analyzed (Bremner and Mulvaney, 1982; Walling et al., 2011). Lentil seeds were decontaminated, and planted in soil after germination (non-sterile soil) with following treatments: (a) the negative control seedlings with no bacterial inoculum (Control, C), (b) seedlings treated with *S. plymuthica* 33GS, and (c) seedlings treated with *Serratia* sp. R6. For cell suspension preparation, the NREs were grown at 28°C ± 2 (O.D._600_ 0.5). A fraction of the collected soil was autoclaved (Debnath et al., 2023). As previously described, lentil seeds were decontaminated, inoculated with the selected NREs and planted in pots with 500 gm of soil. The pots were transferred to a plant growth chamber, set at 13 h of light and 8 h of dark condition, at 25°C, with alternate watering days and reaped after 40 days. The experiment was conducted in triplicate, with each treatment performed in three sets. The number of nodules formed, fresh and dry weight of nodules, root and shoot length, and chlorophyll content were measured (Apha, 1989; Kamble et al., 2015; Debnath et al., 2023). The Kjeldahl method determined the plant’s total nitrogen content (Bremner, 1965). The Leaf relative water content (LRWC) was evaluated with the formula LRWC (%) = [(FW-DW)/TW-DW)]* 100 (Zhang and Blumwald, 2001). FW denotes the fresh weight of the leaf, DW denotes the dry weight of the leaf, and TW denotes the turgid leaf weight. The study was carried out thrice with three replicates.

### 2.10 Detection of root metabolites

Root samples were collected 30 days after inoculation to detect root metabolites. After being treated with liquid nitrogen, the root samples were ground into a fine powder (Isha et al., 2020). The root powder (150 mg) was extracted in 250 ml of 80% methanol using an ultrasonicator (30 min, 40°C). The procedure was repeated twice, and the resulting supernatant was filtered with Whatman filter paper (125 mm). The supernatant was dried and concentrated using a rotatory evaporator before being stored at -80°C for further investigation.

HPLC-grade methanol was used to dissolve the solvent-free root extracts. The samples were analyzed using mass spectrometry and gas chromatography (GC-MS-2010 Plus-Shimadzu). The temperature of the column was initially set at 50°C for four minutes, increased to 320°C at a rate of 7°C per minute, and maintained for 20 minutes. The temperature of the injector was set at 280˚C (split mode = 20:1; injection volume = 0.1 μL), following regulating the carrier gas (Helium) flow to 1 mL/min (total run time = 60 minutes). The mass spectra ranged from m/z 40 to 700, with electron ionization set to 70 eV. The mass spectra of the chromatograms were evaluated against the database of the National Institute of Standards and Technology database library (NIST11).

### 2.11 Rhizospheric community analysis in rice fallow soil using Illumina Miseq sequencing

The soil from the rhizospheric region, derived from *S. plymuthica* 33GS or *Serratia* sp. R6 treatments were collected in sterile containers for analysis of the bacterial community structure. Soil (~0.5 mg) from the proximity of the root was transferred in sterilized cryo-vials placed in liquid nitrogen. The DNA from the soil sample was isolated using the Nucleospin DNA Kit. The amplicon libraries were generated using the Nextera XT Index kit, followed library preparation of 16S metagenomic sequences. 16S rDNA-specific primers, forward: 5’ -GCCTACGGGNGGCWGCAG-3’ and reverse: 5’ -ACTACHVGGGTATCTAATCC-3’, were used to amplify the V3-V4 region. The libraries were sequenced using the 2x300 bp paired-end method on the MiSeq system. Amplicons were amplified with i5 and i7 primers, purified with AMPureXP beads, fluorometrically measured with a Qubit and loaded into Miseq at the appropriate concentration for cluster creation and sequencing. The paired-end sequences were analyzed using the Quantitative Insights into Microbial Ecology (QIIME) software (Price et al., 2010). The sequences were then categorized into operational taxonomic units (OTUs) at 97% sequence similarity, and the phylogenetic abundance and distribution profiles were generated by using UCLUST (Edgar, 2010). Taxonomy was assigned to the OTUs by aligning the reads against the green genes database (version 13-8) (McDonald et al., 2012). The reads with accession numbers SRR11515555 (control), SRR11521673 (rhizospheric soil with *S. plymuthica* 33GS treatment), and SRR11526889 (rhizospheric soil with *Serratia* sp. R6 treatment) were submitted to the NCBI database.

The functions of rhizospheric microbiota were analyzed and predicted for all the treatments and compared with the uninoculated control. The 16S rRNA profile was used to determine the soil samples’ metagenomic function profile and contents. KEGG pathway function was classified at level 3 by the phylogenetic study of communities by reconstructing 222 unobserved states (PICRUSt) program (Langille et al., 2013).

### 2.12 Statistical analysis

The results of all treatments on the lentil plant were described as the average of three independent tests with three replicates. The results are presented as mean ± standard deviation. The bacterial treatment was used as the dependent variable in a one-way analysis of variance (ANOVA), while conditions and parameters were used to determine p ≤0.05 using SPSS (version 20). The Past function (4.03) was used to assess α- diversity values, which combined the Chao-1 approach with the Shannon H and Richness S methodologies. A distance matrix was employed using the Whittaker index from the Past (4.03) to analyze β-diversity. Functional gene analysis was done using the statistical analysis of taxonomic functional profiles (STAMP version 2.1.3). The figures were generated using the ANOVA statistical test and the Tukey Kramer *post hoc* test with p ≤0.95 as the default statistical parameters.

## 3 Results

### 3.1 Isolation and characterization of NREs

A total of 45 NREs were isolated from the root nodules of *L. culinaris*. NREs having multiple PGP traits were selected for further studies. 33GS produced the highest amount of IAA, followed by R6 (108.45 and 107.21 g/ml, respectively). The isolate, 33GS and R6 have an appreciable amount of siderophore activity (77% and 65%, respectively). Also, 33GS and R6 solubilized 240.8 µg/ml by 33GS, and 218.5 µg/ml of phosphate, respectively. R6 showed maximum ACC deaminase activity (1160 nmol α-ketobutyrate mg protein^-1^) h^-1^, followed by the isolate 33GS (580 nmol α-ketobutyrate mg protein^-1^ h^-1^). Hydrogen cyanide (HCN) and ammonia (NH_3_) production were also detected in two isolates, 33GS and R6. Both isolates 33GS and R6 confirmed the presence of *nif* element (*nifH* 360 bp). The sequences were deposited in the NCBI database and accession numbers OQ473647 and OQ473648 has been received for *S. plymuthica* 33GS and *Serratia* sp. R6 respectively.

Based on these results *S.plymuthica* 33GS and *Serratia* sp. R6 were selected for further study (**Table 1**; **Supplementary Figures 2, 3****3**).

**Table 1:** PGP attributes of the isolates.

Attributes	*S. plymuthica* 33GS	*Serratia* sp. R6
Phosphate Solubilization (µg/ml)	240.8 ± 0.02	218.5 ± 0.03
Siderophore (%)	77± 0.07	62 ± 0.38
IAA (µg/ml)	108.45 ± 0.13	107.21 ± 0.06
ACC deaminase (nmol α-ketobutyrate mg. protein^-1^h^1^)	580 ± 0.6	1160 ± 0.01
NH_3_	+	+
HCN	+	+
Potassium solubilization	+	+
EPS production (µg/ ml)	362.9 ± 0.43	331.2 ± 0.18
Biofilm Production (O.D. at 570 nm)	0.89 ± 0.002	0.72 ± 0.002
Protease activity (U/ml)	175.12 ± 0.01	143.23 ± 0.02
Nitrogenase activity (ηmole C_2_H_4_ h^-1^ mg^-1^ protein)	387.6 ± 0.87	315.45 ± 0.79
*nifH* (360 bp)	+	+

*Values given are the mean of three independent observations followed by standard deviation.

### 3.2 Molecular characterization of the NREs

**Figure 2:**
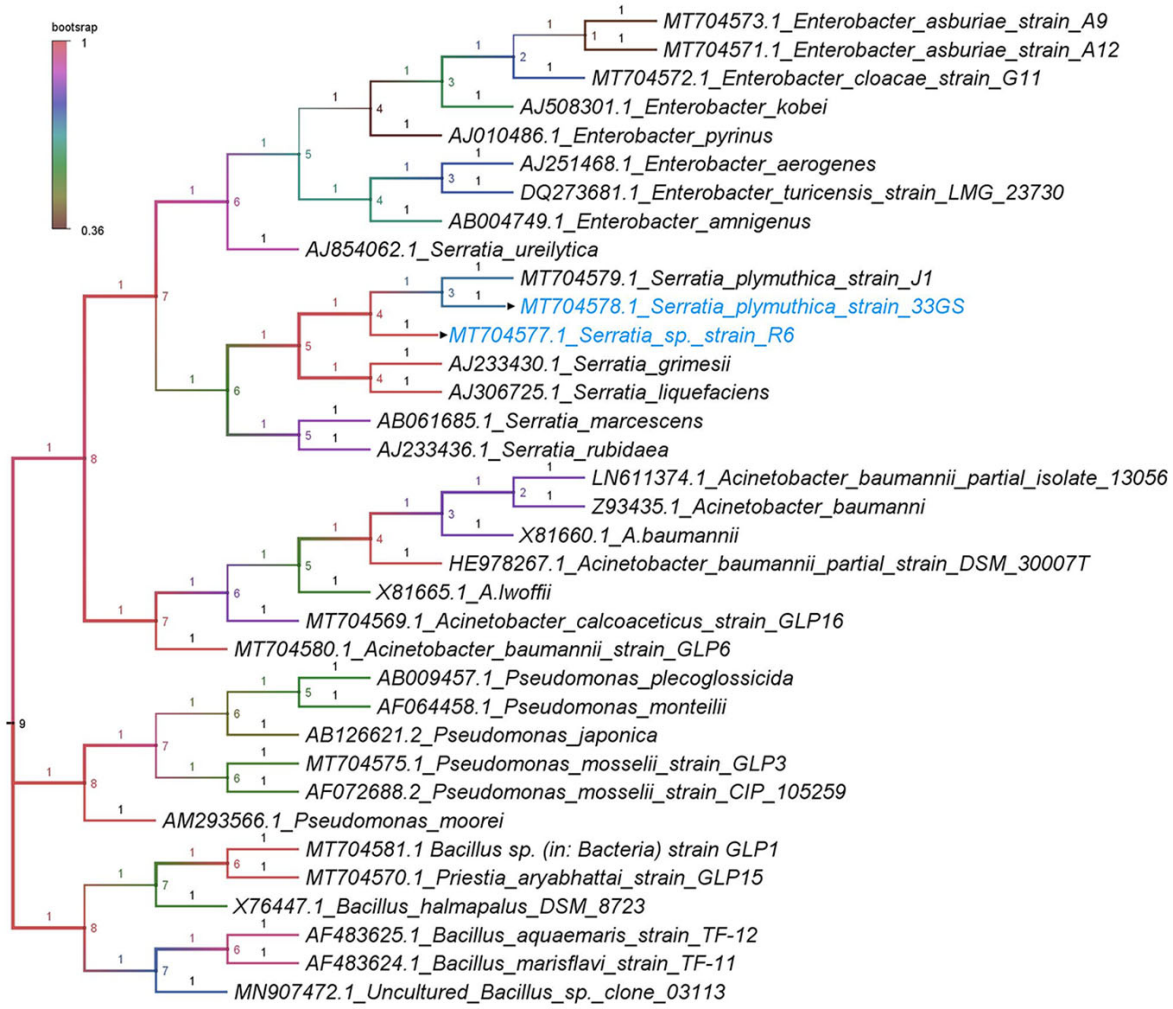
Dendrogram with accession numbers and name of bacteria along with the selected NREs (with arrowheads) as analyzed by the Tamura-Nei model with the neighbourhood-joining method.

The selected NREs were identified as *Serratia* spp. Based on 16S rDNA homology. 33GS was identified as *Serratia plymuthica* (NCBI accession no. MT704578.1), while R6 was not designated up to the species level and was termed *Serratia* sp. R6 (NCBI accession no. MT704577.1). The phylogenetic tree was constructed using the closest relatives of the NREs. The number represents the values at the node of each clade and the colour represents the bootstrap values in a gradient manner (**Figure 2**).

### 3.3 SEM imaging for root hair formation, NRE in the interior of nodules, root surface colonisation, and TEM analysis of the interior of root nodules

**Figure 3:**
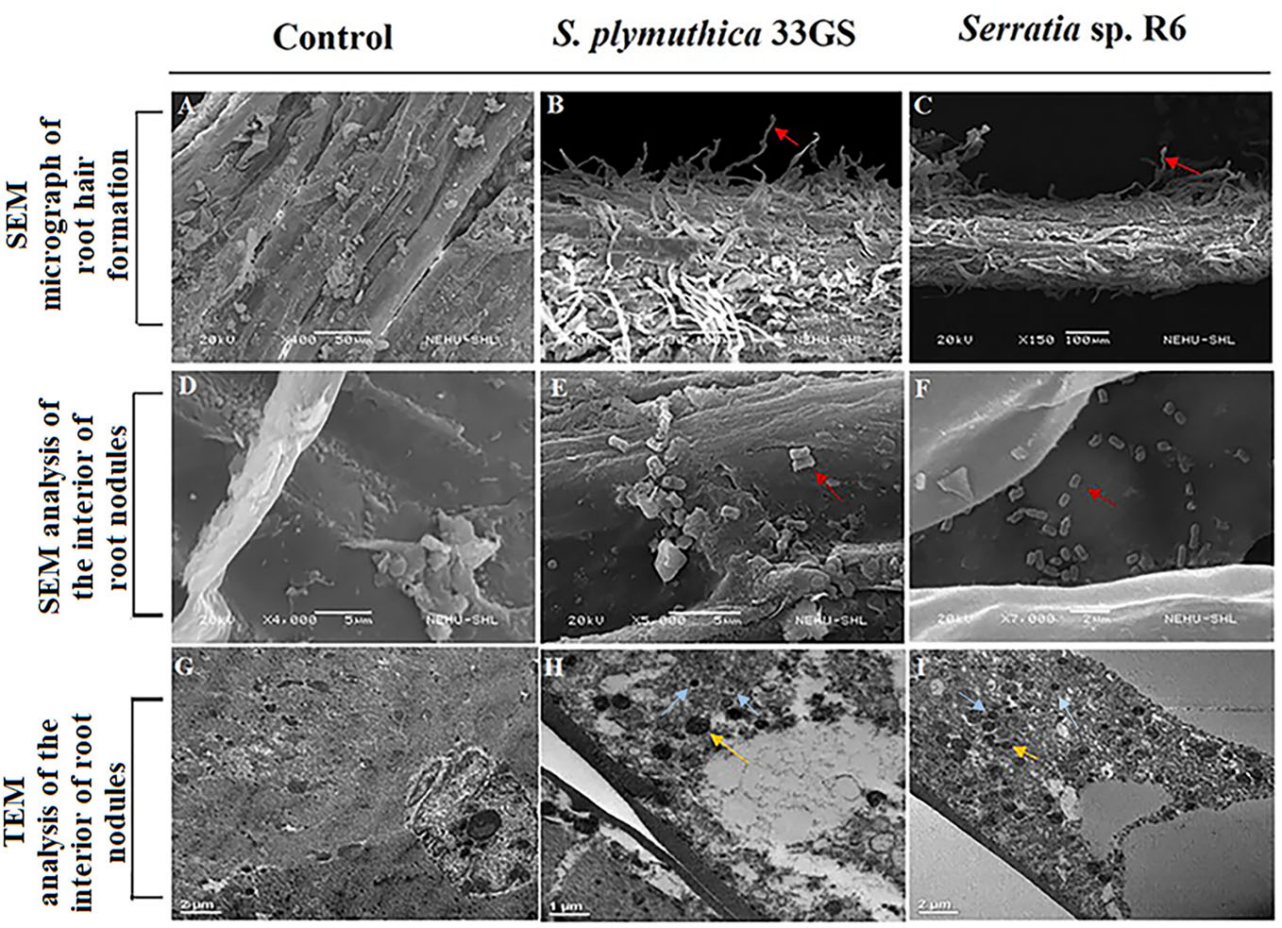
SEM image for root hair formation colonization **(A)** Root of *L. culinaris* without NRE treatment **(B)** Root of *L. culinaris* with *S. plymuthica* 33GS, **(C)** Root of *L. culinaris* with *Serratia* sp. R6 (The root hairs were represented with red arrowheads). SEM image for endophytic colonization of the NREs **(D)** Root nodule of *L. culinaris* without NRE treatment, **(E)** Root nodule of *L. culinaris* with *S. plymuthica* 33GS, **(F)** Root nodule of *L. culinaris* with *Serratia* sp. R6 (The bacterial cells were represented with red arrowheads), TEM image of root nodules **(G)** Control with no bacterial treatment, **(H)** Root nodule of *L. culinaris* with *S. plymuthica* 33GS, and **(I)** Root nodule of *L. culinaris* with *Serratia* sp. R6 (The bacteroids are represented with yellow arrowheads and the NREs are represented with blue arrowheads).

*gfp*-tagged mutants were inoculated with other rhizobial isolates (Debnath et al., 2023) and planted in sterilized soil *via* seed bacterization. SEM imaging of the root surfaces and root interior was done to visualize the microenvironment and tissue topology during colonization, to verify the presence of NRE in the roots and nodules. The *gfp*-tagged cells were re-isolated, from the interior of root nodule issues, and confirmed through partial 16S rRNA analysis. Additionally, we performed TEM analysis to visualize the bacteroid content of the nodules in the treated plants. *S. plymuthica* 33GS and *Serratia* sp. R6 increased root hair formation (**Figures 3A–C**), and colonized the interiors of the root nodules successfully, as evidenced by the SEM imaging (**Figures 3D–F**). The SEM examination of root samples also revealed the cells of *S. plymuthica* 33GS or *Serratia* sp. R6 scattered consistently along the plant root surface as well as inside the root, whereas bacterial cells were not observed with the root samples of the control plant (**Supplementary Figure 4**). The *gfp*-tagged mutants (**Supplementary Figure 5**) were re-isolated from the roots of the treated plants and subsequently checked using 16S rDNA sequencing. Scattered zones of bacteroid growth within the nodules was visible in TEM micrographs. The bacteroids were identified as dark circular areas, which is known to serve as the primary locations for nitrogen fixation within the nodules. Additionally, light-coloured regions were detected, which may be indicative of the presence of granular starch deposits (**Figures 3G–I**). Moreover, it was noticed that number of zone of bacteroids’ growth in the nodules treated with *S. plymuthica* 33GS or *Serratia* sp. R6, were higher as compared to the control group that did not receive any NRE treatments.

### 3.4 Effect of NREs on the growth of lentils with rice fallow soil under greenhouse conditions

Inoculation with both the NREs increased plant’s total nitrogen content, number of nodules and the fresh weight of nodules (in non-sterile soil) (**Table 2**). In the pot experiment (sterile and non-sterile soil), *S. plymuthica* 33GS treatment yielded better results than *Serratia* sp. R6 treatment. The isolates *Serratia* sp. R6 and *S. plymuthica* 33GS positively impacted seedling germination (35 and 29%), and the inoculation of both isolates promoted plant growth (65 and 42%). Further, in comparison to *Serratia* sp. R6, *S. plymuthica* 33GS had a higher germination rate (5%) and better plant growth than the other treatments (**Supplementary Figure 6**). The measure of soil nutrients in NRE-treated plants were evaluated with respect to the untreated plants. The level of potassium was reduced in the NRE-treated soil, particularly in *S. plymuthica* 33GS-treated soil. After applying either isolate, the soil’s available phosphorus content increased significantly. *Serratia* sp. R6-treated soil had more available phosphorus. In addition, the soil that was treated using *S. plymuthica* 33GS possessed the highest amount of soil nitrogen, followed by *Serratia* sp. R6 treatment.

**Table 2:** Effect of NREs treatment on plant growth parameters and soil nutrients.

	Treatments (N=9)	Control		S. plymuthica 33GS		Serratia sp. R6	
	Rice fallow soil	Sterile	Non-sterile	Sterile	Non-sterile	Sterile	Non-Sterile
Plant Parameters	Fresh biomass (g)	0.23 ± 0.06	1.45 ± 0.03a	0.65 ± 0.04b	2.56 ± 0.01c	0.56.7 ± 0.02b	2.13 ± 0.02b
	Dry biomass (g)	0.05 ± 0.04	0.73 ± 0.43a	1.77 ± 0.83a	2.21 ± 0.06b	1.74 ± 0.54a	2.13 ± 0.04b
	Number of nodules	Nil	2 ± 0.86a	Nil	7.23 ± 0.5c	Nil	6.6 ± 0.5d
	Nodule fresh weight (µg)	Nil	6.46 ± 0.17a	Nil	12.54 ± 0.12c	Nil	10.42 ± 0.02c
	Total nitrogen content (%)	0.60 ± 0.005a	1.34 ± 0.004a	1.23 ± 0.007b	1.84 ± 0.004c	1.49 ± 0.01b	1.91 ± 0.04c
*Soil Parameters*	Nitrogen (Kg ha^-1^)	115.32 ± 0.32a	125.44 ± 0.03a	118.04 ± 0.5b	136.54 ± 0.02b	116.66 ± 0.46a	123.16 ± 0.5c
	P (Kg ha^-1^)	5.48 ± 0.2a	5.48 ± 0.2b	5.67 ± 0.48b	8.17 ± 0.03c	5.24 ± 0.45d	10.85 ± 0.07e
	Potassium (Kg ha^-1^)	22.25 ± 0.74a	32 ± 0.35b	22.42 ± 0.19c	28.85 ± 0.03a	22.32 ± 0.09a	30.9 ± 0.26d
	Organic Carbon (%)	0.6 ± 0.01a	0.9± 0.13b	0.65 ± 0.05b	1.2 ± 0.13c	0.63 ± 0.01b	1.15 ± 0.02c
	pH	4.23 ± 0.07a	4.3 ± 0.01c	4.3 ± 0.05c	5.2 ± 0.01d	4.3 ± 0.04b	5.1 ± 0.005c

*Values denoted by different alphabets were significantly different (p ≤ 0.05), while the same alphabets indicate non-significant differences. Values given are the mean of three independent observations followed by the standard deviation.

**Figure 4:**
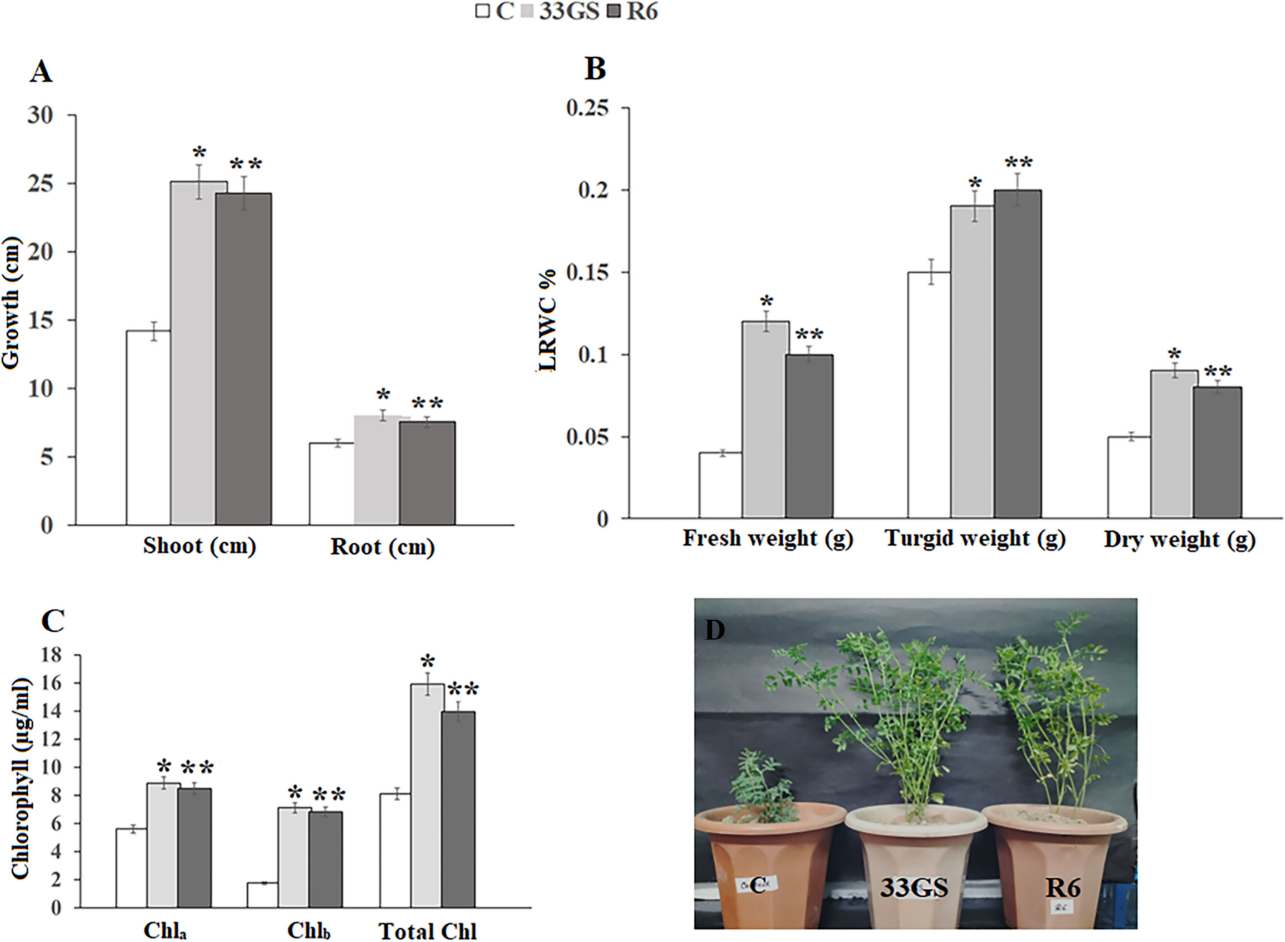
Effect of bacterial treatment on **(A)** root and shoot length of the plant, **(B)** Chlorophyll content of the leaves of all the treated plants, **(C)** Leaf relative water content, **(D)** Growth of lentil plants with uninoculated control, *S. plymuthica* 33GS and *Serratia sp.* R6. Values bearing different signs (*, **, ***) differ significantly (P ≤ 0.05).

The texture of the fallow soil was clayey with nitrogen content 114.12 ± 0.12 (Kg ha^-1^), phosphorous content 5.3 ± 0.13 (Kg ha^-1^), potassium content 20.13 ± 0.54 (Kg ha^-1^), organic carbon 0.57 ± 0.03 (%) and soil pH 4.13 ± 0.02. Furthermore, the pH of the NRE-treated soil samples was marginally higher than that of the untreated soil samples. In comparison to the untreated control, the levels of organic carbon were higher in NRE-treated soil samples, with *S. plymuthica* 33GS treatment having the highest amount of organic carbon (**Table 2**). In contrast to the untreated plants, the NREs increased the length of shoots and roots and chlorophyll levels in the leaves. Also, the water retention in leaves was higher (**Figure 4**; **Supplementary Figure 7**).

### 3.5 Detection of root metabolites

**Figure 5:**
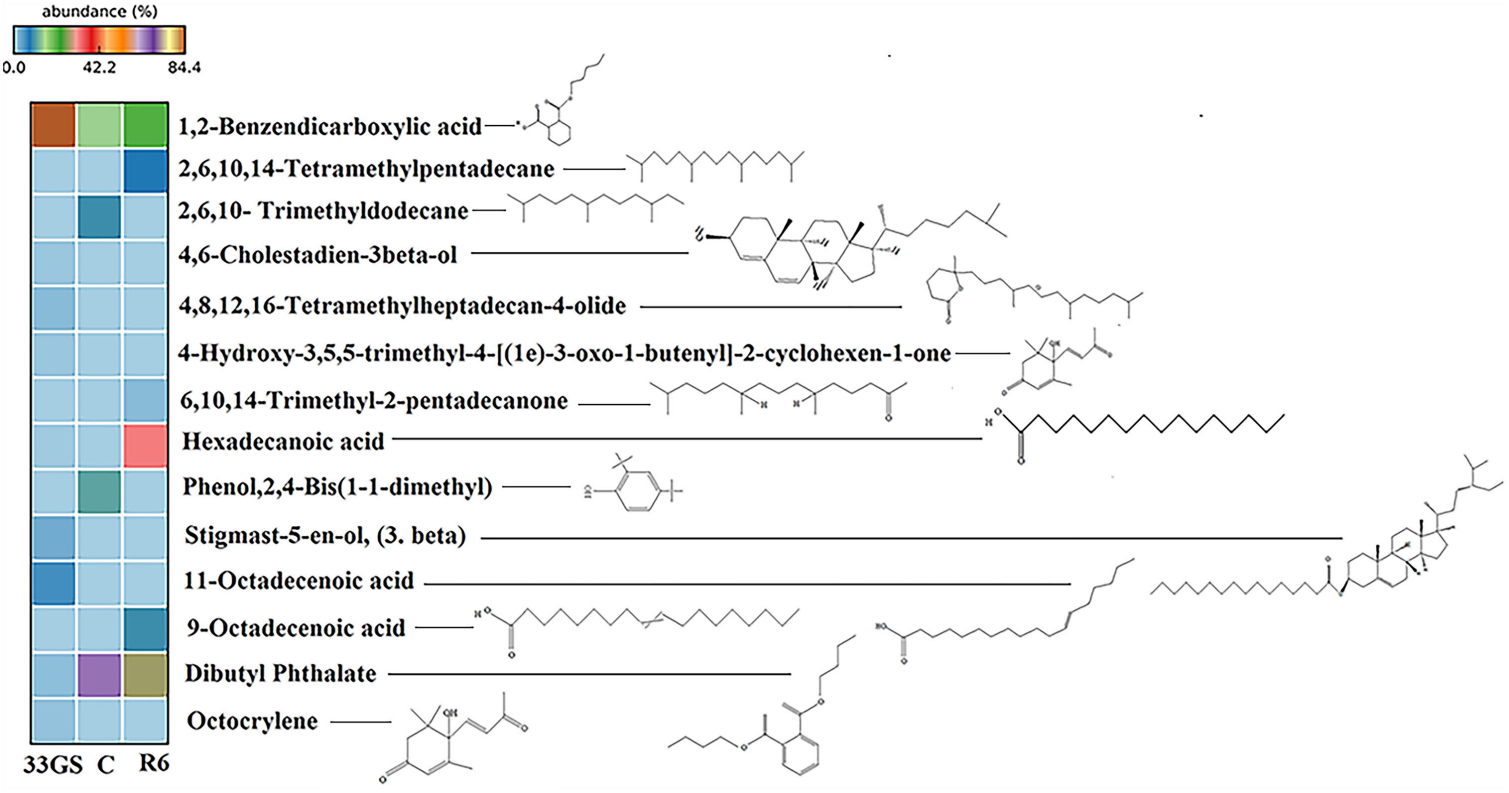
Heat map showing the distribution of root metabolites in all the treatments. Each heat map row represents metabolite detection in different treatments with colour patterns relative to the abundance of the respective compound.

13 organic compounds were detected and identified in the GC-MS profile of lentil root metabolites after 30 days of treatment with either *S. plymuthica* 33GS or *Serratia* sp. R6. Phenol,2,4-Bis(1,1-Dimethyl), Dodecane,2,6,10-Trimethyl, was detected only in the control treatment without NRE inoculation. The treatment with *S. plymuthica* 33GS resulted in metabolites such as Methyl-4-[(1e)-3-Oxo-1-Butenyl]-2-C, 11-Octadecenoic Acid, and octocrylene. With *Serratia* sp. R6 treatment, 2-Pentadecanoe, 6,10,14- Trimethyl, 9-Octadecenoic Acid (Z), 4,8,12,16-Tetramethylheptadecan-4-Olide and Cholesta-4,6-Dien-3-Ol,(3.Beta) were detected. Dibutyl Phthalate and 1,2-Benzene Dicarboxylic Acid were detected in root metabolites of all three treatments. In contrast, Hexadecanoic Acid, Methyl Ester was found only in the root of plants with bacterial treatments *S. plymuthica* 33GS and *Serratia* sp. R6 (**Figure 5**; **Supplementary Figure 8**)

### 3.6 Variation in rhizospheric microbiota of lentil under the influence of NREs in a typical fallow soil

**Figure 6:**
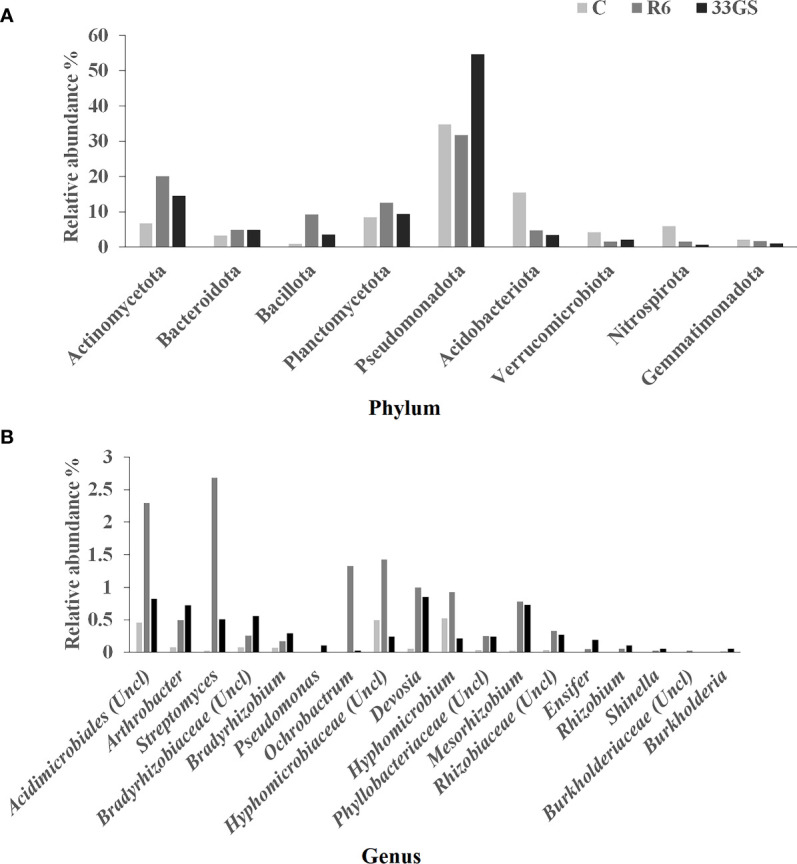
Relative abundance (%) of the rhizospheric microbiota of lentil **(A)** Phylum level, and **(B)** Genus level in different treatments (Control, *S. plymuthica* 33GS and *Serratia* sp. R6).

Pseudomonadota dominated the phylum level in the uninoculated control, followed by Acidobacteriota, Planctomycetota, and Actinomycetota. However, when the respective NRE was inoculated, the bacterial population differed from the uninoculated control. After treatment with *S. plymuthica* 33GS, there was an increase in abundance in Pseudomonadota (20%), Actinomycetota (8%), Bacillota (3%), Planctomycetota (1%), and Bacteroidota (1%). While *Serratia* sp. R6 treatment increased the abundance of Actinomycetota (14%), Bacillota (9%), Planctomycetota (4%), and Bacteroidota (1%) (**Figure 6A**).

Alpha-proteobacteria were found to be the most prevalent class, preceded by Beta- and Delta-proteobacteria in the untreated control. There was an increase in abundance of Actinobacteria (9%), Alphaproteobacteria (5%), Planctomycetia (5%), and Cytophagia (0.9%) upon inoculation of *S. plymuthica* 33GS. Similarly, the treatment with *Serratia* sp. R6 enhanced the relative abundance of Actinobacteria (12%), Alphaproteobacteria (9%), Planctomycetia (5%), and Cytophagia (2%) with the addition of. However, in comparison with the untreated control, both NRE inoculation reduced the relative abundance of Betaproteobacteria. (**Supplementary Figure 9A**).

As observed, Rhizobiales were the most abundant order level, followed by Xanthomonadales, Sphingomonadales and Actinomycetales. The population of the following were seen to be elevated after the *S. plymuthica* 33GS treatment Rhizobial (1%), Actinomycetales (12%), Bacillales (1.9%), and Xanthomonadales (4%), whereas *Serratia* sp. R6 treatment increased Rhizobiales (6%), Actinomycetales (12%), Bacillales (4%), and Xanthomonadales (2%) (**Supplementary Figure 9B**).

Further, treatment with either of the two NREs enhanced the abundance of some of the beneficial genera compared with the uninoculated control. *Rhizobium*, *Mesorhizobium*, *Bradyrhizobium*, *Streptomyces*, *Ochrobactrum*, *Devosia*, *Arthrobacter*, *Ensifer* and *Burkholderia* were in higher abundance in bacterial-treated soil samples (**Figure 6B**).

**Figure 7:**
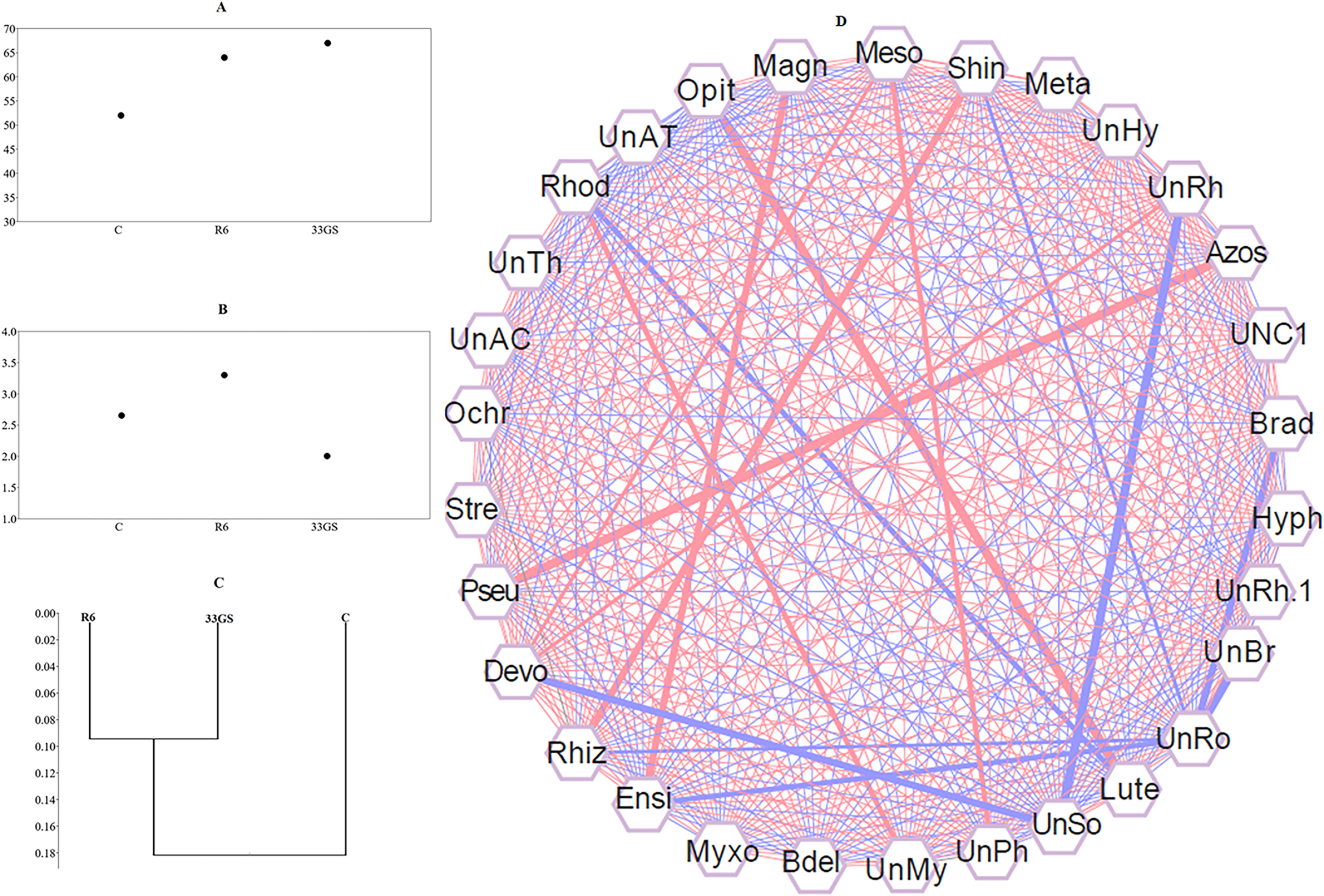
**(A)** Shannon-H index, **(B)** Chao 1 index, **(C)** Beta diversity analysis of the samples using the Whittaker index, distance matrix, and **(D)** Correlation analysis exhibiting the degree of association between bacteria genera. The width of the lines between the nodes indicates the strength (P ≤ 0.05). The richness of associated taxa is correlated positively with red lines, and negatively with blue lines. Cytoscape (Version 3.9) was used to visualize the network.

Assessment of alpha diversity was done using a rarefaction curve used to quantify the diversity of the bacterial population, which measures species diversity based on the specific amplified sequence variants (ASVs) found in a specific sample. This study demonstrated that *S. plymuthica* 33GS inoculation enhanced the number of bacterial populations (**Supplementary Figure S8**). α-diversity varied considerably among the treatments and control (**Figures 6A, B**). However, for the β- diversity, the two treatments were almost similar to the uninoculated control (**Figure 7C**).

The relative abundances of various bacterial genera in all the treatments were correlated to determine the cooperation of the various interdependent bacterial genera and the impact on the dynamics of the bacterial population. This unique co-occurrence phenomenon was evaluated using Pearson’s correlation coefficient (P< 0.05). The interconnections between the bacterial populations are shown by the nodes and edges. The red lines exhibit strong, more beneficial associations between bacterial groupings, while the blue lines indicate a negative relationship (**Figure 7D**). In the analysis, a specific set of bacteria demonstrated a significant positive association, illustrating their interdependence. Both NRE treatments increased the abundance of some bacterial genera along with their corresponding genera. A strong correlation was observed between the abundance of *Azospirillum* (Azos) and *Pseudomonas* (Pseu)*, Shinella* (Shin) and *Rhizobium* (Rhiz*)*, *Opitutus* (Opit) and *Luteolibacter* (Lute)*, Magnetospirillum* (Magn) and *Ensifer* (Ensi), *Mesorhizobium* (Meso) and *Devosia* (Devo), *Mesorhizobium* (Meso) and an unclassified genus of *Phyllobacteriaceae* (UnPh), *Devosia* (Devo) and Unclassified genus *Bacteriaceae* (UnRh), and *Rhodoplanes* (Rhod) and an unclassified *Myxococcales* (UnMy), etc. Moreover, a negative correlation was observed between *Rhizobium* (Rhiz) and an uncharacterized genus of *Rhodospirillaceae* (UnRo), *Devosia* (Devo) and an uncharacterized genus of Solirubrobacterales (Unso), *Ensifer* (Ensi) and an unclassified genus of *Rhodospirillaceae* (UnRo), *Rhodoplanes* (Rhod) and *Luteolibacter* (Lute), and between few other genera (**Figure 7**).

### 3.7 Prediction of functional genes of the rhizospheric microbiota

**Figure 8:**
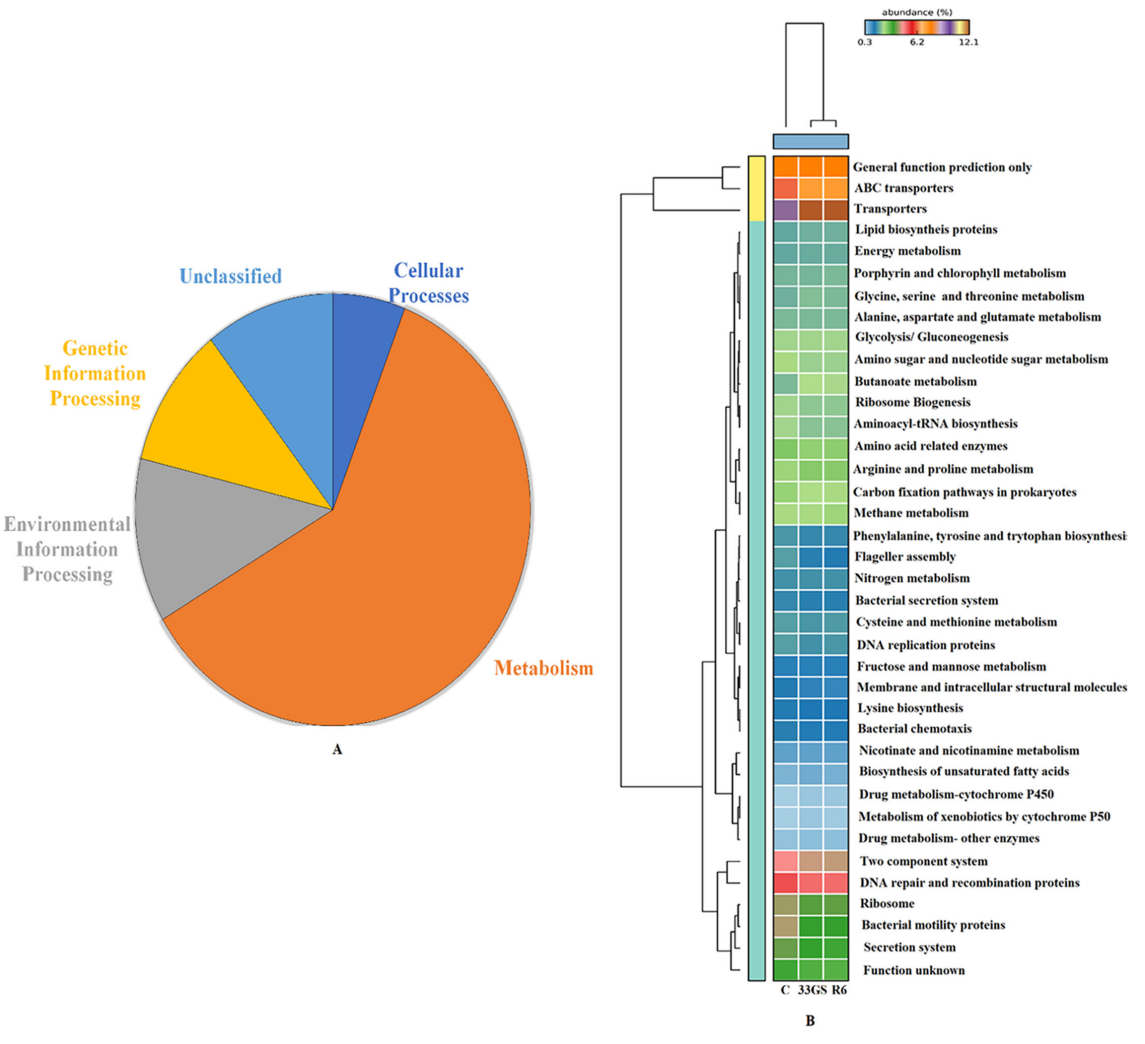
**(A, B)** Heatmap of the samples (KEGG level 1 and 2 analysis).

**Figure 9:**
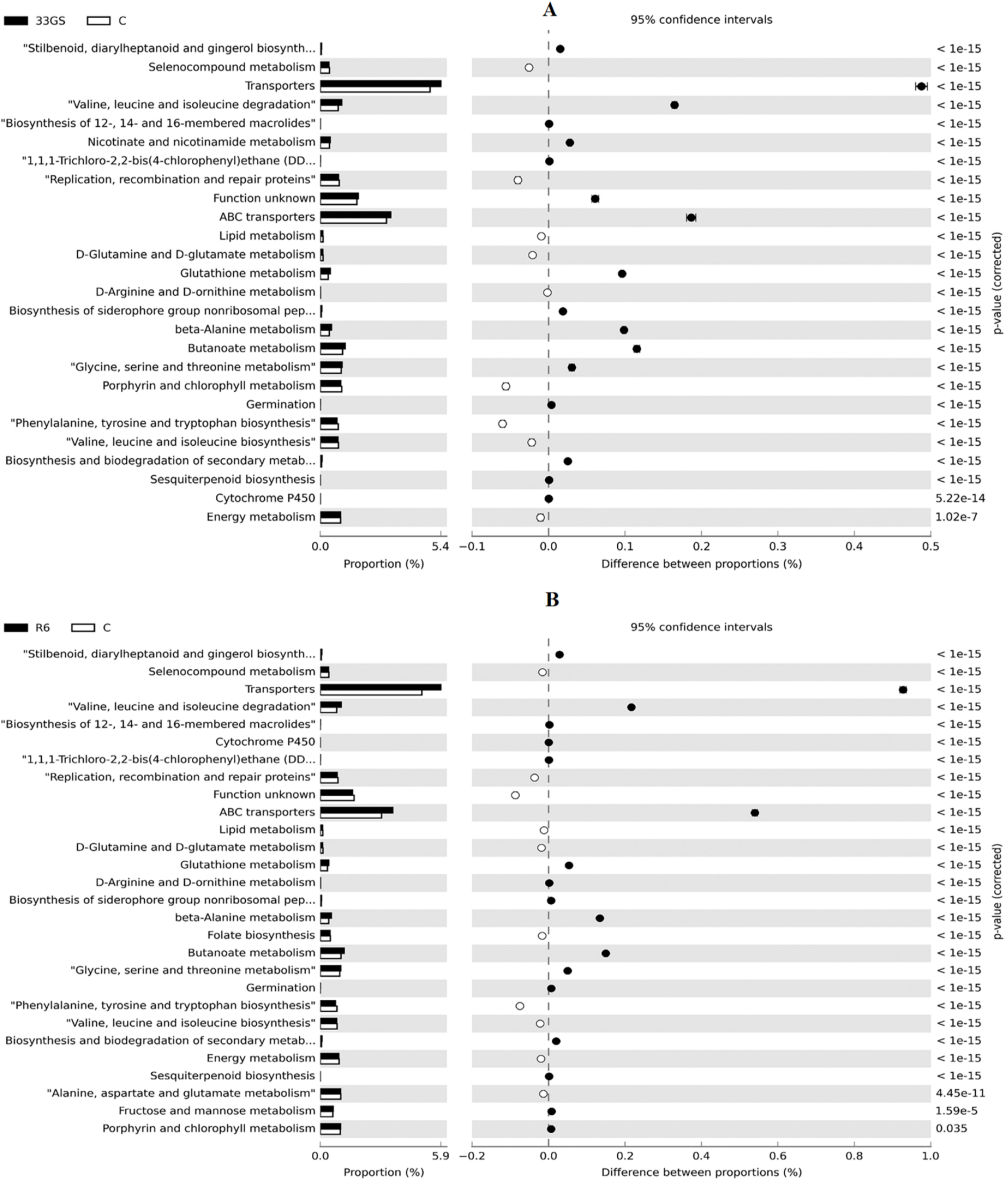
**(A, B)** Relative abundance of genes related to various functions of untreated control with NRE-treatments **(A)** *S. plymuthica* 33GS and **(B)** *Serratia* sp. R6.

The microbial community’s functional aspects were predicted and annotated using KEGG pathway analysis. As per KEGG Level 1 observation, the abundance of genes in the NRE-treated samples differs substantially. The treatment with NREs had an impact on five major functional categories i.e., cellular functions, environmental information processing, metabolism, and genetic information processing (**Figure 8**). At KEGG level 3, The abundance of genes associated with processes such as glutathione metabolism, carbohydrate metabolism, tryptophan metabolism, nicotinate and nicotinamide metabolism, biosynthesis of 12-, 14-, and 16-member macrolides and phosphotransferase system (PTS) was projected to be significantly high in both bacterial treatments when in comparison to the untreated control (**Figure 9**).

## 4 Discussion

The root nodules constitute distinct environments formed in roots of the leguminous plant’s systems because of the highly specific symbiotic relation between NREs and legumes. The aim of this research was to investigate the root nodules of *L. culinaris* plants in various agricultural fields in Assam, India. The study demonstrates that NREs are efficient plant growth promoters that also contribute to the enhancement of plant growth, in the presence of other symbiotic root-nodule bacteria (Muresu et al., 2019; Deng et al., 2020; Tokgöz et al., 2020). Previously, we have reported the potential rhizobial candidates from the lentils’ root nodules, which could increase plant growth (Debnath et al., 2023).

Here, we have isolated forty-five culturable NRE bacteria from root nodules of lentils, and among these, thirteen PGP isolates were characterized using 16S rRNA sequence homology. Most of these isolates were *Enterobacter* and *Serratia*, while few were *Bacillus* and *Pseudomonas*. The NRE bacteria demonstrated different PGP traits, which include siderophore, phosphate solubilization, nitrogen fixation, potassium solubilization, IAA, HCN, and ACC deaminase, which is in accordance with the previous reports of NREs with different host plants such as *Vigna radiata* (Bhutani et al., 2018; Sansanwal et al., 2018; Mowafy et al., 2022), *Glycine max* (Zhao et al., 2018), and *Pisum sativum* (Maheshwari et al., 2020).

Although the selected NREs did not stimulate nodulation on their own in sterilized soil, they could improve nodulation and growth of lentil plants when paired with other rhizobial bacteria present in non-sterile soil. In the greenhouse experiment, both *S. plymuthica* 33GS and *Serratia* sp. R6 positively affected nodulation (non-sterile soil conditions). Furthermore, in both sterile and non-sterile conditions, NRE-treated plants performed better in total nitrogen content. It is generally believed that the production of root nodules is associated with better fixation of atmospheric nitrogen for plants (Kobayashi et al., 2021), which was evident in the plant’s total nitrogen content. Moreover, the NREs’ nitrogen-fixing ability was validated by ARA along with the presence of *nifH* gene that has a role in nitrogen fixation (Chowdhury et al., 2009; Delmont et al., 2018).

Enhanced root and shoot length or healthy plants are usually correlated with increased leaf water retention capacity and chlorophyll content (Pavlović et al., 2015). According to Bezawada et al. (2013) and Hanif et al. (2020), various *Serratia* species have been found to produce EPS and biofilms that promote plant growth. A longitudinal SEM scan revealed that the selected NREs colonized intracellular and intercellular gaps, penetrating the inner compartments of the roots, thus confirming endophytic colonization. An adaptable bacteria might enter a plant *via* the tips of roots and hairs or small openings brought on by various factors in the epidermal tissues (Compant et al., 2008; Hardoim et al., 2015). Previous reports have shown that *Serratia* can colonize certain crops, such as rice plants (Gyaneshwar et al., 2001, Cao et al., 2009). There aren’t any reports of *Serratia* colonizing the roots of lentils or other legumes.

Root metabolites had recently been realized for their role in shaping the rhizospheric community (Hu et al., 2018). Also, specific bacteria like *Bacillus subtilis* has been reported to modulate root exudate composition of tomato plants (Korenblum et al., 2020), by the process termed as SIREN (systematically induced root exudation of metabolites). Both of these interrelated phenomena were observed, where the application of NREs modulated the root exudate composition, and henceforth, the rhizospheric bacterial community was also modified. Compounds such as fatty acids, their methyl esters, isoprenoid and triterpenes, were detected from the root of lentil plants. Fatty acids and isoprenoids could potentially have a role in suppressing pathogens (Zhou et al., 2011; Isha et al., 2020; Lange et al., 2020). Pentadecane, 2,6,10,14- Tetramethyl-1-(Methylsulfonyl) is an isoprenoid which has been reported to play an active part in plant development (Vranová et al., 2012). Here, hexadecanoic acid, octadecadienoic, 1,2-benzene dicarboxylic acid, 4-hydroxy-3,5,5-trimethyl-4-[(1e)-3-oxo-1-butenyl]-2-cyclohexen-1-one, octocrylene, 4,6-cholestadien-3beta-ol, and stigmast-5-en-3-ol, (3. beta.) were detected in the NRE-treated plant roots. Among these, 1,2-benzene dicarboxylic acid, hexadecenoic acid, and octadecadienoic acid are known to possess antimicrobial properties (Sekar et al., 2018). Moreover, hexadecenoic acid and octadecadienoic acid have been reported to alter soil microbial dynamics (Bi et al., 2021). Also, the triterpenes has been reported to modulate the microbial community structure (Pascale et al., 2020) by regulating the endophytic microbes’ ability to accumulate metabolites and improve plant health (You et al., 2021). The triterpenes detected here were 4,6-cholestadien-3beta-ol and stigmast-5-en-3-ol (3.beta) in the *S. plymuthica* 33GS inoculated plant roots. The application of NREs, enhanced the relative abundance of Bacteroidetes, while Deltaproteobacteria was considerably reduced in numbers. A similar observation has been reported by Pascale et al. (2020) upon the detection of triterpenes in the roots of the *Arabidopsis* plant (Huang et al., 2019). Besides, 4,6-cholestadien-3beta-ol has biocontrol properties and has been reported previously from *Serratia* sp. inoculated plants (Zhou et al., 2011; Zhu et al., 2018), whereas stigmast-5-en-3-ol (3. Beta) acts as a plant defence molecule and a growth regulator (Faure et al., 2009) and has been reported for controlling the fluidity of plant membranes for adaptation to temperature changes (Piironen et al., 2000). Dibutyl Phthalate was consistently present in all the treatments. It is a bio-control agent that can alter the microbiome and related functions (Wang et al., 2016; Yang et al., 2016). The primary aromatic root metabolite detected uniformly in all three treatments was 1,2-benzene dicarboxylic acid, which was reported for its allelopathic properties by providing plant stability (Duan et al., 2020).

A significant aspect of understanding microorganisms’ fundamental mechanisms is studying the complex relationships between plants and their rhizospheric microbiota. It shows their capabilities to improve plant growth and modify the microbiome’s structure for better yield. The microbiome in the rhizosphere consists of a distinct, taxonomically organized population of microbes. These microorganisms build multiple arrays of relationships involving host plants, resulting in beneficial, commensal or pathogenic traits. The effectiveness of the inoculum depends upon specific interactions between the bacteria (host) and the plant. A good bacterium colonizes the plant’s root and rhizosphere while promoting its growth with its multiple PGP. Additionally, the bacterial inoculum modulates the rhizospheric microbiota (Hu et al., 2020). However, an inoculated strain’s efficiency and capacity to thrive in new environments or communities are also essential factors in a successful interaction (Piromyou et al., 2011; Touceda-González et al., 2015). Xu et al. (2020) suggested treatment with useful microbes may result in host plant-dependent microbiome shifting may result in host plant-dependent microbiome shifting, also known as microbiome engineering (Baudoin et al., 2009; Jeong et al., 2013; Marwa et al., 2020; Singha and Pandey, 2021) and resulting alteration of microbiota inside a very short period (Kong et al., 2019). Therefore, adding the NREs could help in establishing synergistic or symbiotic relationships, which then help in adapting and proliferating different microbes in the soil. Inoculation with the selected NREs significantly enhanced the length of root and shoot, as compared to the uninoculated control. Rawat et al. (2020) also has reiterated that plants’ growth depends on the rhizospheric interaction of microorganisms.

Low productivity results from rice fallows that have poor soil properties and fertility. Gangwar et al. (2006) suggested that legumes be grown as a secondary cultivar to improve the condition of the soil. Here, in this study, the soil nutrient status was improved after adding *L. culinaris* and NREs. The rhizospheric soil of NRE-treated plants had a significantly higher N, P, K, organic carbon content along with better growth of lentils. Pratibha et al. (1996) noticed elevated production of legumes in rice fallows during the dry season, resulting in improved drought tolerance and N accessibility (Gan et al., 2015). Sowing leguminous plants as a secondary cultivar are known to improve soil enzymatic activity and microbial population (Gautam et al., 2021). The NREs-treated soil had a relatively higher pH, which had previously been linked to an increase in Bacillota (Anderson et al., 2018). As previously suggested, it can also be linked to an increase in Bacteroidota and a reduction in Acidobacteriota in the NRE-treated soil (Zhang et al., 2017). Due to the increasing use of pollutants and other chemicals, agricultural soils have grown acidic and become less productive over time. According to Yuan et al. (2019), the plant’s growth may have resulted in a decrease in Chlorobiota in the rhizosphere, indicating healthy soil. The upsurge in Planctomycetota in the lentil rhizosphere is in accordance to earlier finding (Pramanik et al., 2020), implying that specific root exudates are involved (Qiao et al., 2017). The role of the rhizospheric microflora in plant growth is crucial. The root microbiome is shaped by interactions involving plants and microbes (Xu et al., 2020). These bacteria possess multi-PGP properties that recruit potential bacterial communities that promote plants’ growth. The rhizospheric microbiota can be altered by modifying plant’s metabolism by changing the native microflora (del Carmen Orozco-Mosqueda et al., 2018; Kotoky et al., 2018; Arif et al., 2020).

Inoculation with *S. plymuthica* 33GS or *Serratia* sp. R6 shaped positive associations between multiple species. The correlation data showed that the NRE treatments impacted a group of bacteria whose interdependence was vital for several key functioning, thereby supporting the plant’s strength. The genera important for these functions are either missing or in limited numbers in the untreated control. The use of NREs stimulated plant development and transformed the rhizospheric microbial community, suggesting that *Serratia* sp. R6 or *S. plymuthica* 33GS inoculation significantly impacts the bacterial population in the rhizosphere, which is also affected by the below-ground interactions (Korir et al., 2017). The NREs bacterial treatment attracted mutually dependent groups of bacteria that were otherwise relatively less abundant in the uninoculated control. In fact, the bacterial community in lentil rhizosphere soils is complex and diverse, and it contains microorganisms that can work in a mutualistic, symbiotic, or pathogenic manner with the lentil plant, and also can be manipulated with bacterial augmentation (Kong et al., 2019). Depending on the kind of interaction with the plant, these bacteria may be advantageous or detrimental (Ye et al., 2022). In this study, the rhizospheric soil treated with NRE had a higher relative abundance of bacterial phyla such as Bacillota, Actinomycetota, Planctomycetota, and Bacteroidota. In addition, the uninoculated control had a lesser abundance of *Arthrobacter, Mesorhizobium, Rhizobium, Devosia*, *Bradyrhizobium*, *Ensife*r*, Ochrobactrum*, *Pseudomonas*, and *Streptomyces*. The network analysis revealed that certain nodes with a significant correlation of relative abundance, were part of a cluster containing soil with high pH levels, NPK, and organic matter. Therefore, it’s justified to infer that microbial interactions and environmental factors might have supported the persistence of beneficial bacteria in the rhizosphere, as also observed earlier in a different study (Debnath et al., 2023). Similarly, in another report, Yang et al. (2022) observed an increase in interdependent groups of bacteria with high pH values. Also, Guo et al. (2022), observed that NPK fertilisers resulted in an increase in the abundance of several beneficial taxa.

In comparison to the untreated control, the NRE treatment also exhibited differences in the abundance of genes involved in glutathione metabolism, ABC transporters, germination, siderophore biosynthesis, sporulation, and non-ribosomal peptides. According to Karpiński (2019), macrolides suppress various pathogens, including fungi. Additionally, the soil was procured from a typical rice fallow under which synthetic fertilizers had been used, which might result in an adversative effect on functions like non-ribosomal peptide biosynthesis and siderophore group in the untreated control (Kotoky and Pandey, 2020), but which were improved with bacterial treatment. This study found fewer nitrogen-fixing genes in the rhizospheric region compared to the uninoculated control. According to Sohn et al. (2021), nitrogen-fixing genes are more prevalent in the plant’s later stages.

## 5 Conclusion

The results confirmed the potential of NREs for lentil growth promotion. The treatment with the NREs, i.e., *S. plymuthica* 33GS and *Serratia* sp. R6 distinctively modulated the root exudates pattern in lentil plants and specifically induced the secretion of hexadecanoic acid, octadecadienoic, 1,2-benzene dicarboxylic acid, 4-hydroxy-3,5,5-trimethyl-4-[(1e)-3-oxo-1-butenyl]-2-cyclohexen-1-one, octocrylene, 4,6-cholestadien-3beta-ol, and stigmast-5-en-3-ol. Furthermore, our data exhibited the role of NREs as modulators of the rhizospheric community structure. The data of this research suggests the possibility of a strategy that involves the multipartite interaction of NREs, rhizospheric microbiota, rhizobia and plants with a potential application in rice fallow soil for improving soil health and enhanced growth of lentils.

## Data availability statement

The datasets presented in this study can be found in online repositories. The names of the repository/repositories and accession number(s) can be found in the article/**Supplementary Material**.

## Author contributions

SD performed the experiments, prepared the draft manuscript and is the major contributor. SC and ML, helped with the data analysis. KC, VA, AS, PR, AT, and DM helped in preparation of the manuscript. PP designed and supervised the research and also finalized the manuscript. All authors contributed to the article and approved the submitted version.
