# Supplementary figures and images for: Non-rhizobial nodule endophytes improve nodulation, change root exudation pattern and promote the growth of lentil, for prospective application in fallow soil
Source: Front Plant Sci. 2023 Apr 11;14:1152875. doi: 10.3389/fpls.2023.1152875 (PMC10126288; doi:10.3389/fpls.2023.1152875)

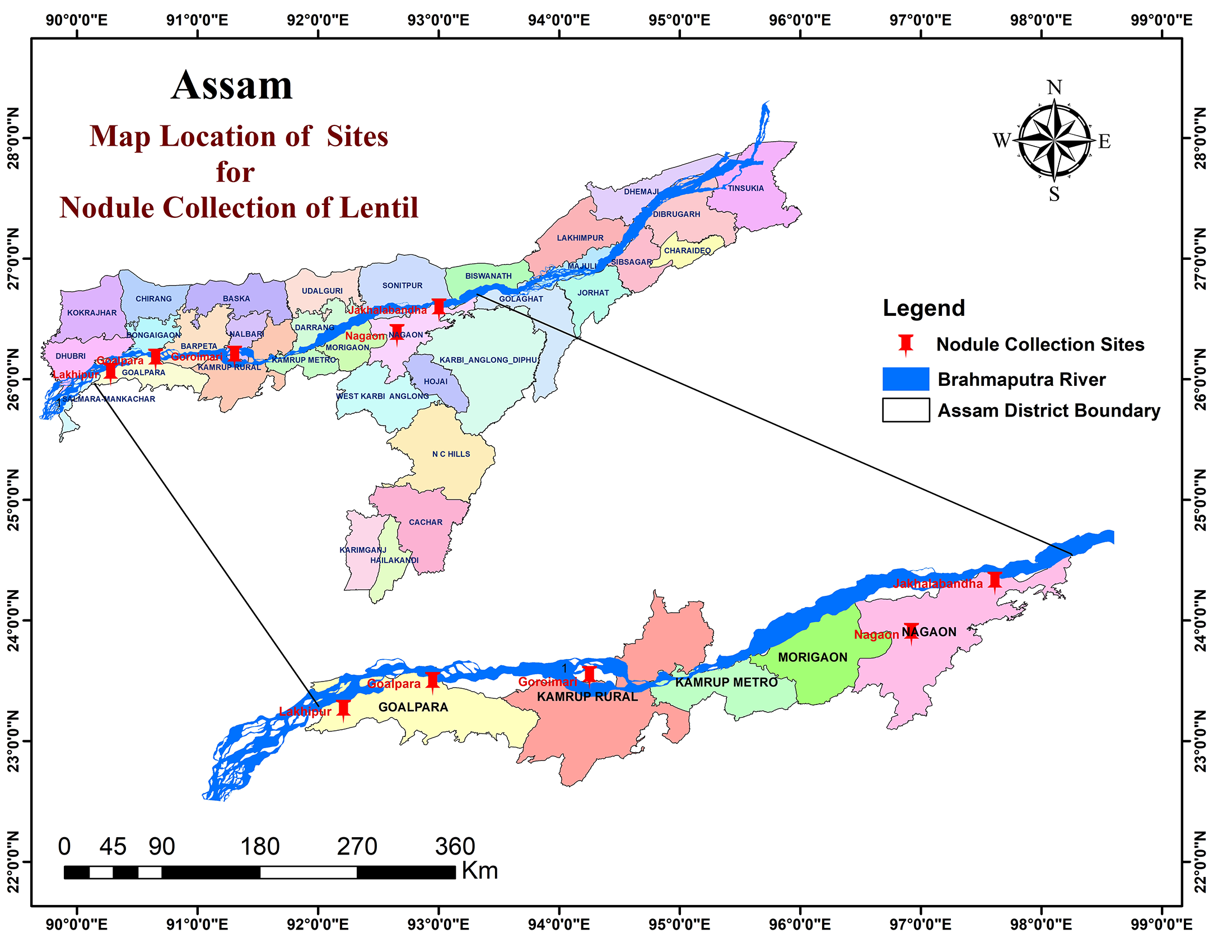

Supplement: Supplementary file 1 [file Image_1.tif]

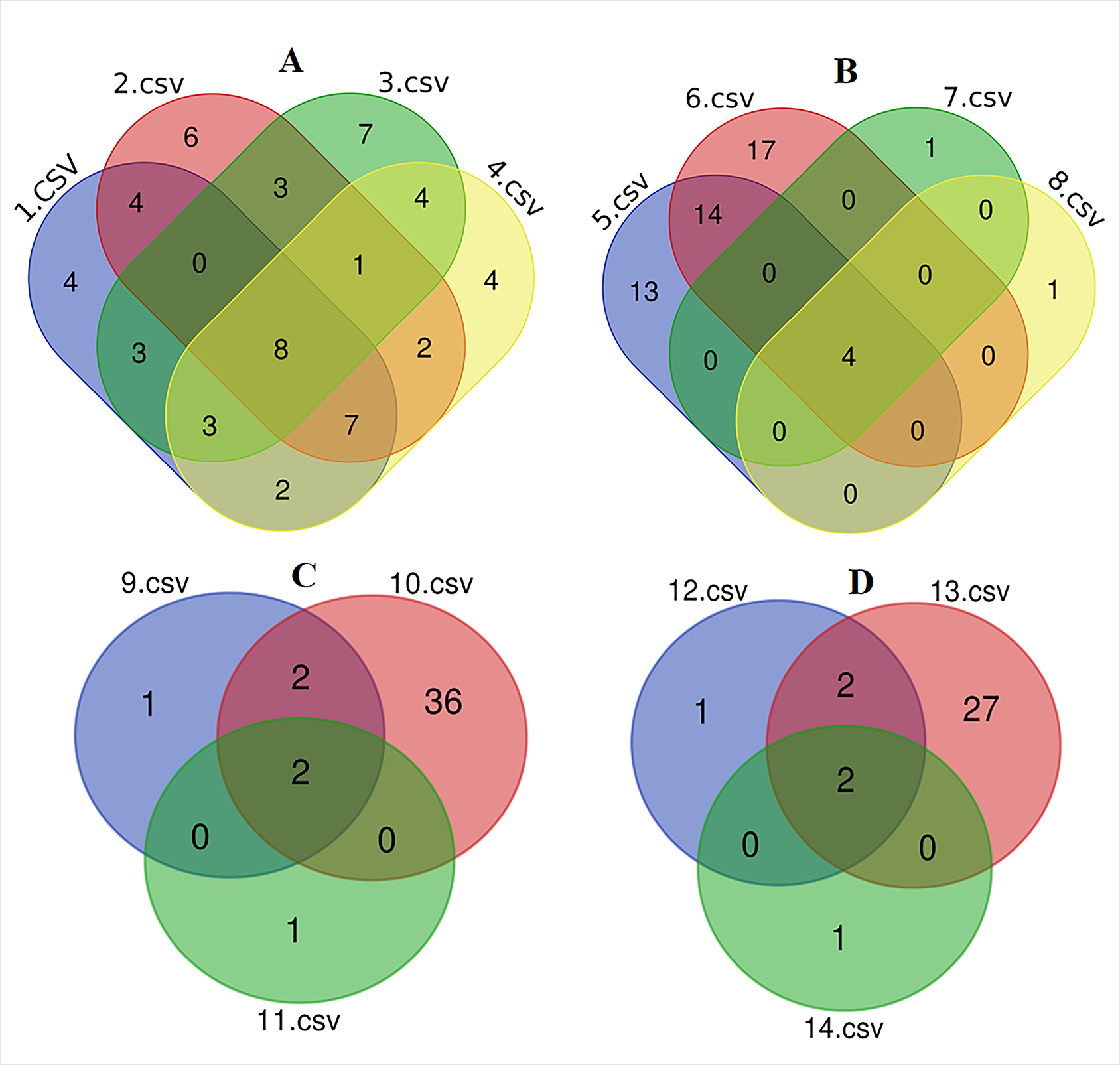

Supplement: Supplementary file 2 [file Image_2.tif]

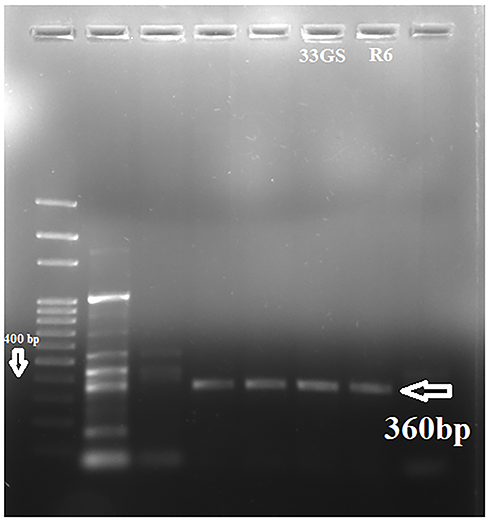

Supplement: Supplementary file 3 [file Image_3.tif]

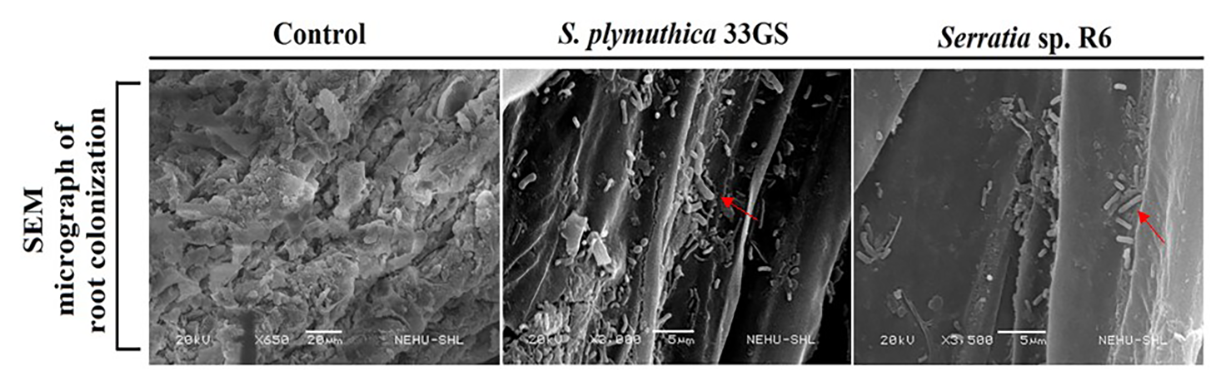

Supplement: Supplementary file 4 [file Image_4.tif]

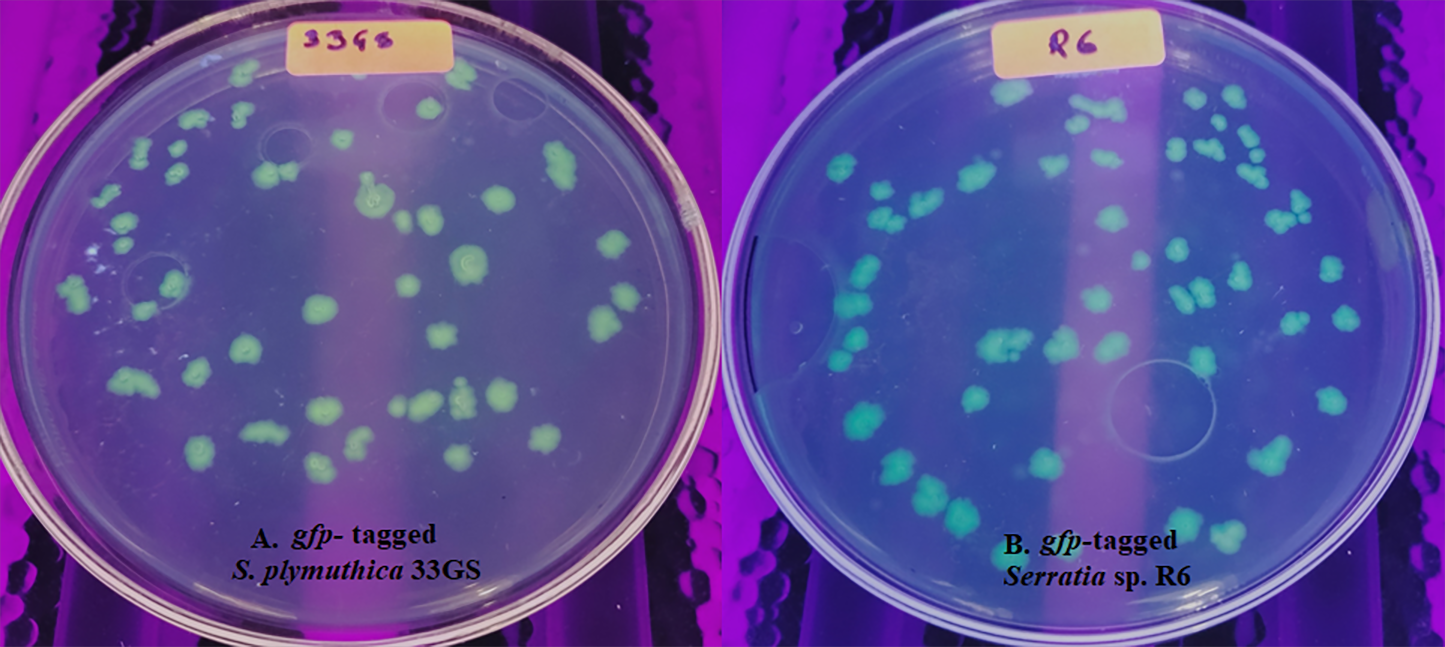

Supplement: Supplementary file 5 [file Image_5.tif]

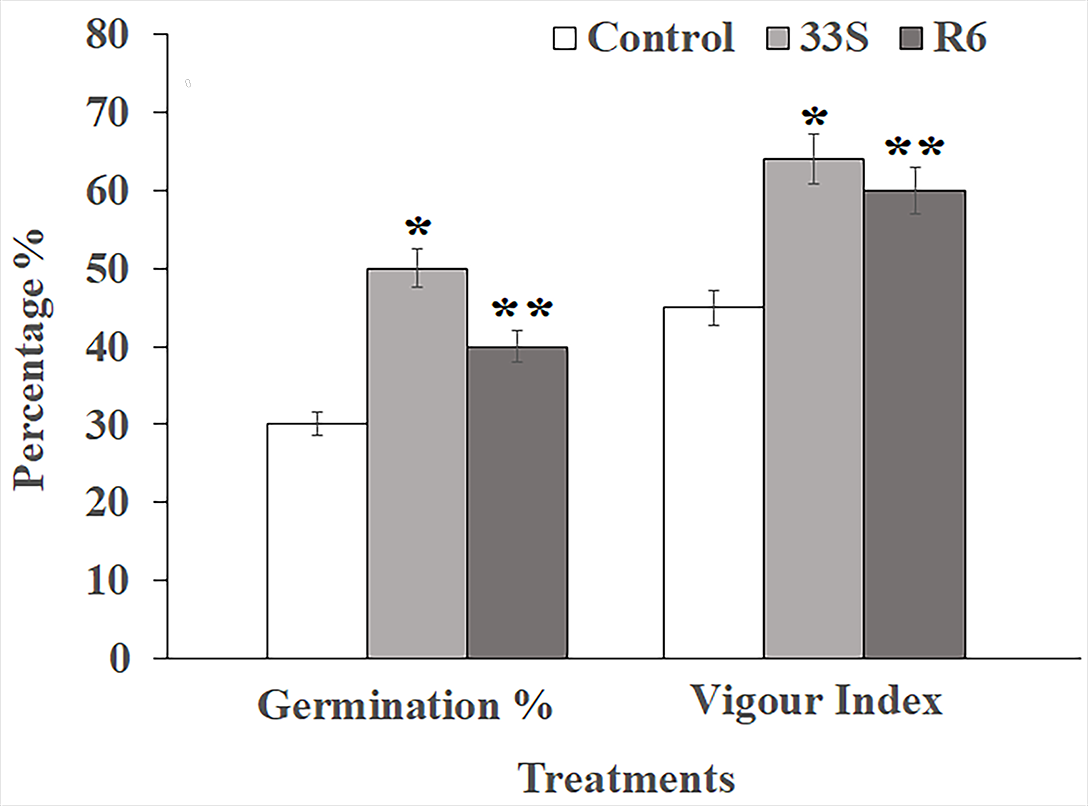

Supplement: Supplementary file 6 [file Image_6.tif]

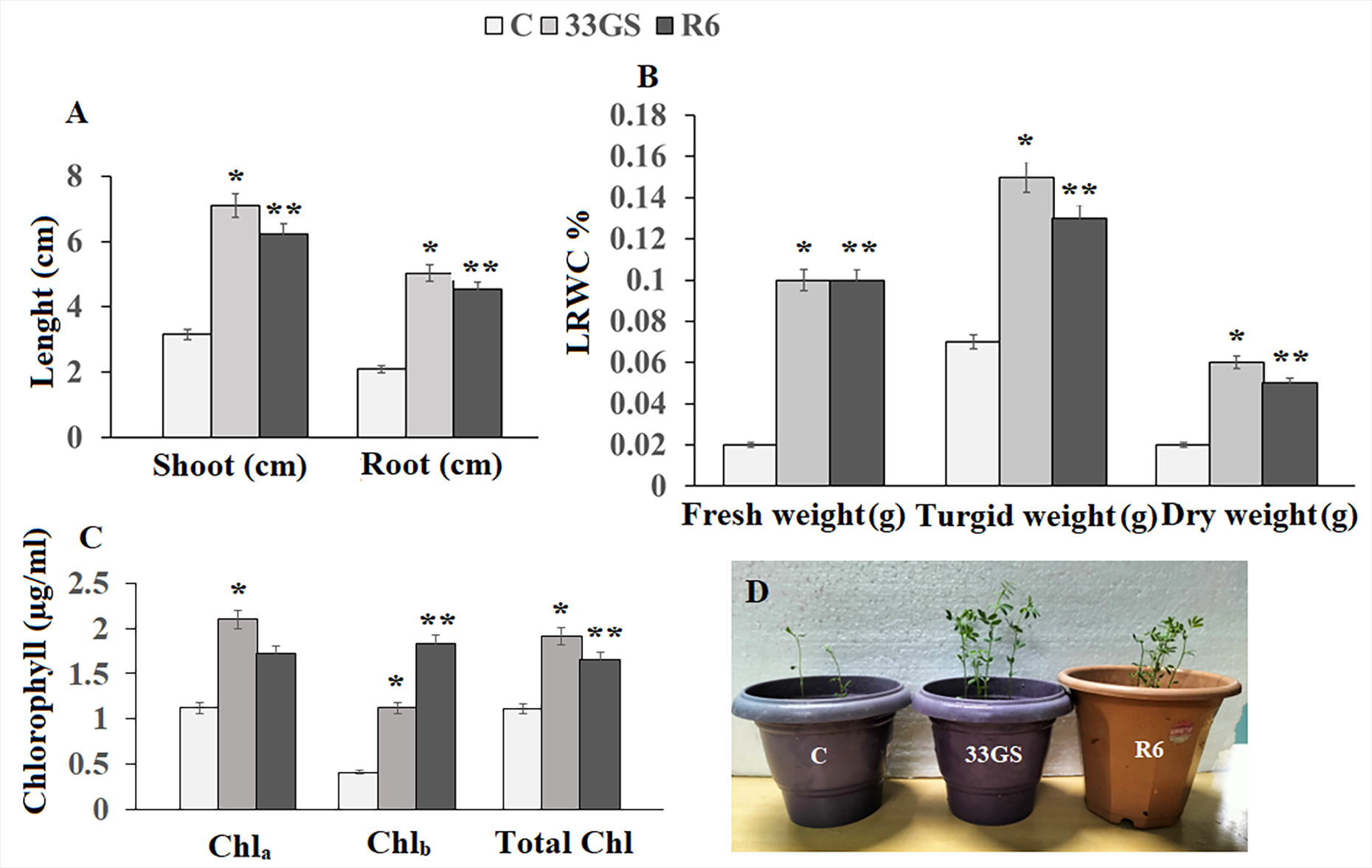

Supplement: Supplementary file 7 [file Image_7.tif]

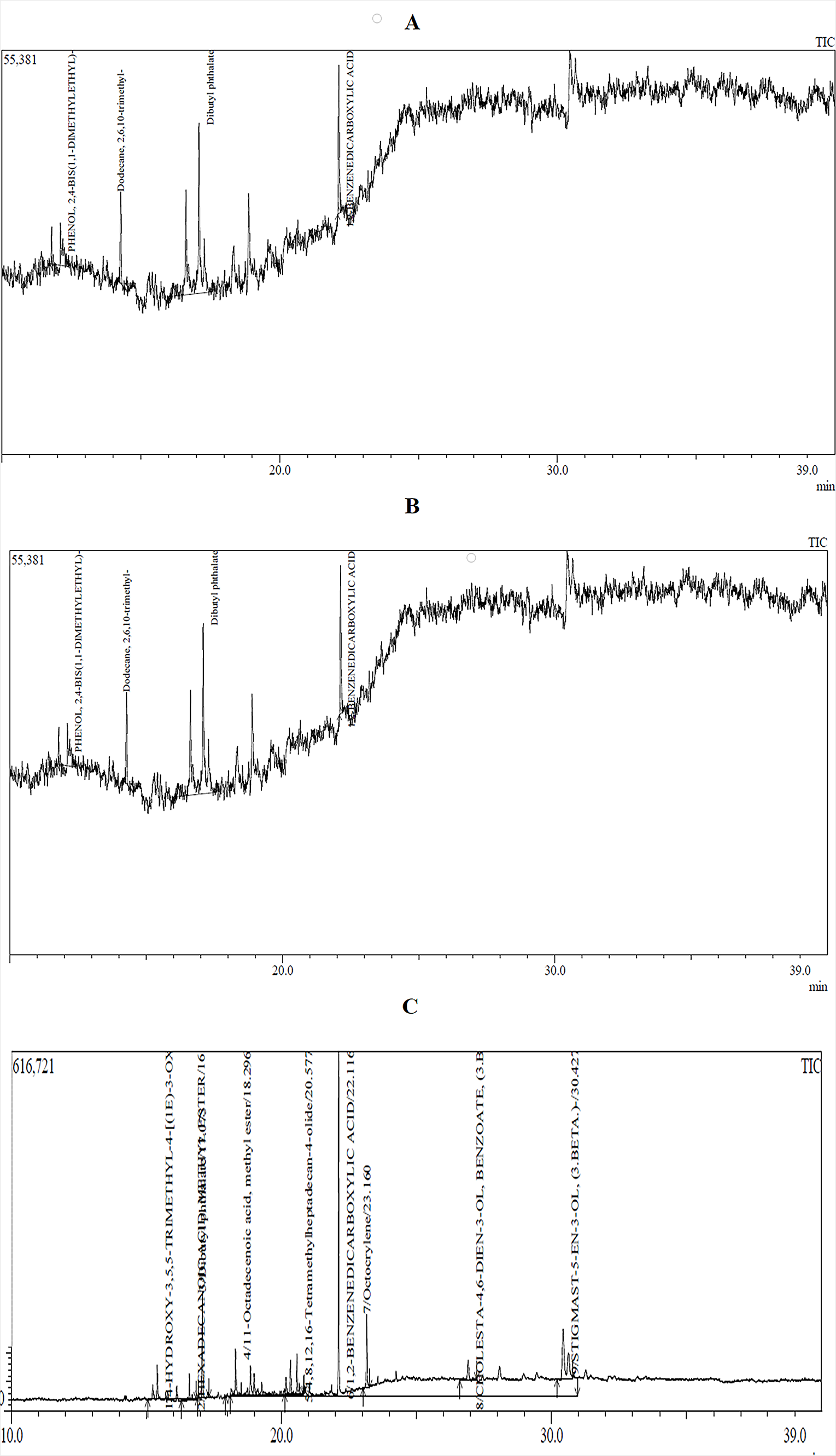

Supplement: Supplementary file 8 [file Image_8.tif]

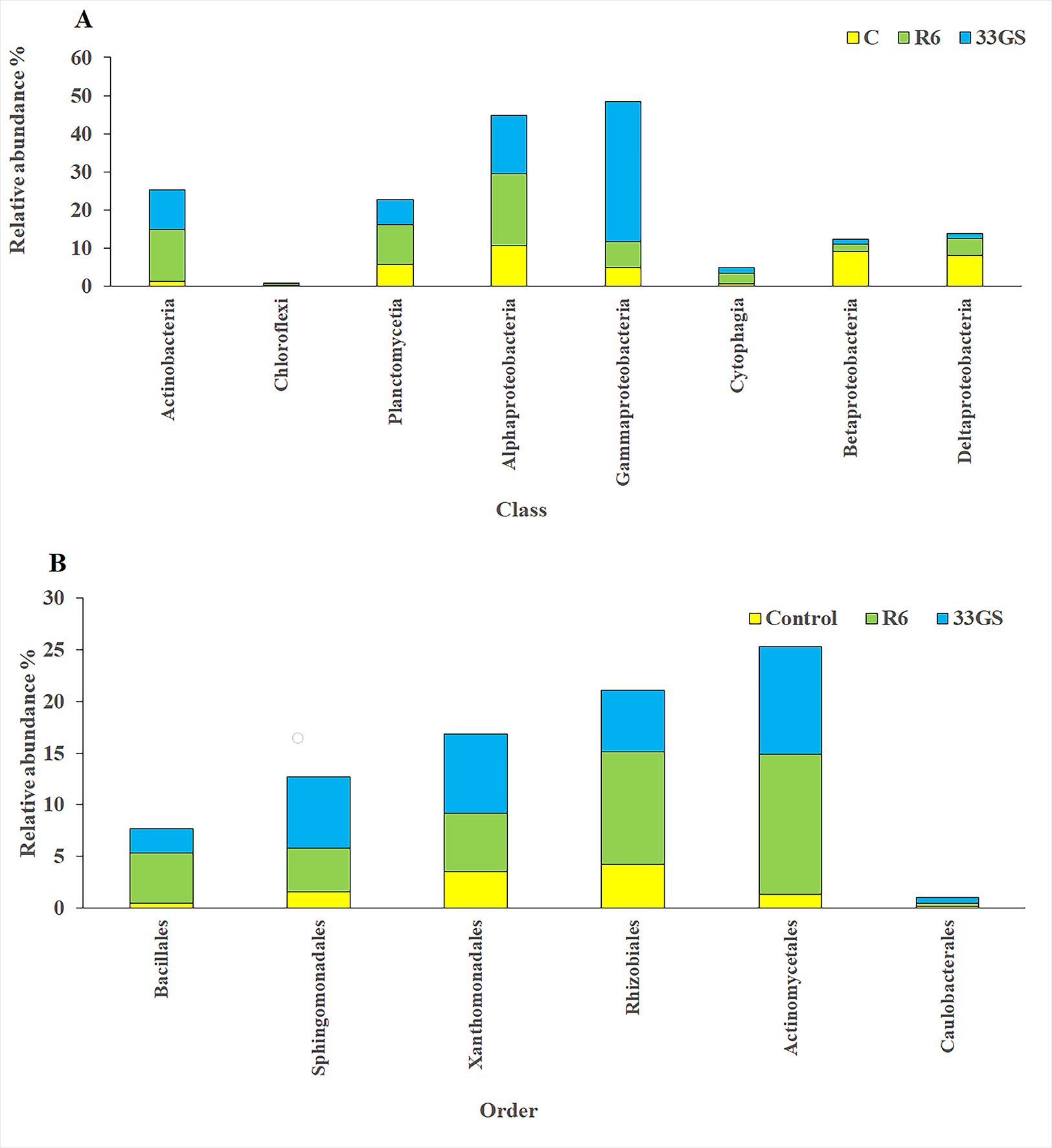

Supplement: Supplementary file 9 [file Image_9.tif]
